# Supplementary material for: Reaching Syrian migrants through Dutch municipal registries for hepatitis B and C point-of-care testing
Source: PLoS One. 2025 Jan 17;20(1):e0316726. doi: 10.1371/journal.pone.0316726 (PMC11741604; doi:10.1371/journal.pone.0316726)
Supplement: Attachment — Submitted filename: Rvwr Cmts_Reply.pdf [file pone.0316726.s003.pdf]

PLOS ONE

Reaching Syrian migrants through Dutch municipal registries for hepatitis B and C point-of-care testing

--Manuscript Draft--

**Manuscript Number:**	PONE-D-23-37923R1
**Article Type:**	Research Article
**Full Title:**	Reaching Syrian migrants through Dutch municipal registries for hepatitis B and C point-of-care testing
**Short Title:**	Hepatitis B and C screening among Syrian migrants in the Netherlands
**Corresponding Author:**	Chrissy Moonen, MSc<br>Maastricht University Care and Public Health Research Institute: Universiteit Maastricht Care and Public Health Research Institute<br>Maastricht, Limburg NETHERLANDS, KINGDOM OF THE
**Keywords:**	Hepatitis B, Hepatitis C, Point-of-Care Testing, Emigrants and Immigrants, Diagnostic Screening Programs
**Abstract:**	Undetected chronic hepatitis B virus (HBV) and hepatitis C virus (HCV) infections can lead to cirrhosis and liver cancer. Syrian migrants are the largest non-European migrant group in the Netherlands with HBV and HCV prevalence rates above 2%. This study aimed to reach Syrian migrants for HBV and HCV screening using point-of-care testing. A multifaceted strategy was employed to reach Syrian migrants aged ≥16 years from two Dutch municipalities for free-of-charge HBsAg and anti-HCV point-of-care testing (POCT) using finger prick blood at the regional Public Health Service. All were personally invited by the Public Health Service by postal mail, based on municipal registry data. Respondents' medical history data were analysed descriptively and data on age, sex, and municipality were compared with non-participating invitees, using Pearson's Chi-square test. Of the study population (N=832), 32.3% (n=269) attended the screening. The mean age of participants was 36 years (range 16-70), 59% were men, and 66.5% were unemployed. Non-participation was higher in the younger age groups (<30 years) (p <.001). The POCT using finger prick blood was well received. None tested HBsAg or anti-HCV positive. With approximately one-third participation, this study demonstrated successful reach of Syrian migrants for screening after a multifaceted strategy. However, while the reach could be considered successful, screening failed to demonstrate new infection in this key population. Thereby, other methods may be preferred to identify new HBV and HCV infections, such as opportunistic screening within existing care processes.
**Order of Authors:**	Chrissy P.B. Moonen, MSc
	Elfi E.G.H. Brouwers
	Christian J.P.A. Hoebe
	Nicole H.T.M. Dukers-Muijrers
	Jamila Bouchaara
	Inge H.M. van Loo
	Casper D.J. den Heijer
**Response to Reviewers:**	Dear Editorial Office and reviewers,<br><br>We thank you for considering our manuscript and for your positive and valuable comments. The revised manuscript, with track changes, has been resubmitted. This document contains the response to comments.<br><br>Reviewer 1<br><br>This well written manuscript describes a study among Syrian migrants in two Dutch cities. The aim was to develop an outreach strategy to screen them by measuring antibodies in the blood, against hepatitis B virus (HBV) and hepatitis C virus (HCV).

The Syrian migrants were chosen because it was suspected that these infections were more highly prevalent than among other Dutch residents.
I have some minor comments to further improve the manuscript.

Comment 1. In the introduction two aims are stated (lines 74-75): 1. how to best reach the Syrian migrants in two Dutch municipalities and 2. To identify HBV and HCV infections in these migrants. For the second aim I suggest to add everywhere in the manuscript that finger prick blood is used next to using a POCT since these two are not coupled and both are deviant from routine testing settings.
Reply: Thank you for your positive feedback and valuable comments. ‘’using finger prick blood’’ has been added throughout the manuscript after POCT (line 33, 77, 84, 236, 299-300, 335, 392).

Abstract
Comment 2. Line 26: please add ‘(POCT)’ after ‘testing’, since this abbreviation is used later in line 32.
Reply: Thank you for the thoughtfulness. (POCT) has been added after testing (line 26).

Comment 3. It is also good to add ‘using finger prick blood’ after ‘POCT’.
Reply: We agree with this suggestion. ‘using finger prick blood’ has been added (line 26).

Multifaceted strategy
Comment 4. Line 127: since there are very many POCT on the market, I suggest to replace ‘the’ by ‘a’ before ‘point-of-care testing’.
Reply: Thank you for your suggestion. We have removed 'we' from the sentence but did not replace it with anything (line 130). Using 'a' would create an awkward combination with the abbreviation, hence our choice to leave out both.

Results
Comment 5. Legend of Fig 2. (line 220): I suggest to add ‘and test results’
Reply: We share your view and have added ‘and test results’ (line 223).

Comment 6. Line 234: Please provide some more information on ‘short turnaround time’: what was the median time and range in minutes?
Reply: Test results were read after 20 minutes. We did not keep exact track of how quickly the doctor communicated the results, but it was immediately after. Therefore, we added, ‘of approximately 20 minutes’ (line 238).

Discussion
Comment 7. Line 300: please adjust and do not start a new sentence. So ‘was made, for example….’.
Reply: We have adopted this suggestion (line 305).

Comment 8. Line 303: please adjust to: ‘non-participants’.
Reply: Thank you for your suggestion. We have adjusted ‘non-participators’ to ‘non-participants’ (line 309).

Comment 9. Line 326: Please replace ‘whom’ by ‘who’.
Reply: This has been adjusted in the manuscript (line 331).

Comment 10. Line 332: Please adjust to ‘successful’.
Reply: Thank you, this has been revised (line 337).

Limitations
Comment 11. Although the DETERMINE test for HBsAg and the OraQuick assay for HCV antibody detection are both stated to have a very high sensitivity, would you still consider using these in future settings on finger prick blood?
Reply: Thank you for your question. These tests were easy to administer and all tests were performed successfully, so yes we would consider using these in future settings on finger prick blood. However, we acknowledge that the tests do not provide information on passed HBV infections, so no statements can be made about this within this population. Although we already covered this very briefly, we have elaborated a

little more on this: ‘’Nevertheless, we lack certainty due to non-participation and lack of information about their cleared HBV infections hepatitis status., as the HBV test did not provide information on this.’’ (line 310-311).

Comment 12. Also, I think it is a pity that no tests seem to have been performed concomittantly on regular EDTA blood samples (as was described to be performed in case confirmation was needed) and in the finger prick blood samples with the POCTs, to have an impression of the actual validity of the high sensitivity of these POCTs in this setting.
Reply: We agree with this observation. Ideally, we would confirm the test results, but for budgetary reasons, we could not do a validity study alongside it.

Reviewer 2.

In this manuscript, the authors aimed to reach Syrian migrants as part of a nationally coordinated implementation research to reach first-generation non-European migrants for HBV and HCV screening using point-of-care testing in two Dutch municipalities (Heerlen and Maastricht). The article described a solid and very well-presented research which is absolutely consonant with OMS agenda for viral hepatitis elimination by 2030.
Overall, the study had great merit in the detailed presentation of the multifaceted strategies used that made it possible to reach a significant number of migrants (approximately 1/3 of the individuals invited to participate), an extremely valuable information that could be implemented in similar studies in different parts of the world in an effort to achieve the microelimination of viral hepatitis in key populations. Although previous studies had reported HBV and HCV infection rates over 2% in Syrian migrants, no positive cases were detected within the 269 individuals screened. Nonetheless, based on a previous systematic literature review that supported the multifaceted strategies proposed by the authors, the research efforts fulfilled their objective of screening a difficult-to-reach population, addressing one of the main bottlenecks for the elimination of viral hepatitis.

The quality of the written English is excellent thorough out the article and, in my opinion, only a few minor revisions are necessary for publication:

Comment 1. Page 13, line 277: A parenthesis ")" mistakenly appeared before the beginning of the sentence.
Reply: Thank you for your observant eye. The parenthesis is deleted (line 281).

Comment 2. Page 13, line 278: An "n" was missing before “=3” in the parenthesis showing the number of people who reported lack of knowledge about hepatitis.
Reply: Thank you, this has been added (line 282).

Comment 3. Page 13, line 291: There is an error in the caption of figure 3: part(non)participation is written instead of (non)participation.
Reply: Again, thank you very much for paying attention to these textual errors. ‘part’ has been deleted (line 295).

Comment 4. Page 15, lines 331 and 333: I believe the in-text citations of references 34 and 35 are not correctly presented and should be corrected.
Reply: We share your view and have adapted these in-text citations (line 336 and line 339), and also applied this to line 329.

Additional Editor Comments:

Dear Author,

	I have read the paper and comments of the reviewers. After careful reading, I observed that sampling strategy adopted by the authors is not so good, the optimal alternative should be RDS, Starfish, etc. However, authors should explain why they choose this sampling method, they may show that a less than optimal technical choice could be better once the relative costs are taking in consideration. Please also read and answered the reviewer's comments.<br><br>Sincerely,<br><br>Livia<br><br>Response: Dear Editor, thank you very much for reviewing our manuscript and for your observations. We value the thoughtful comments provided by you and the reviewers. To arrive at an appropriate sampling strategy for this population, focus groups were conducted. We elaborate on this in lines 104-110: ‘‘A Syrian physician employed by the PHS undertook focus groups with Syrian migrants, conducting separate sessions for men and women. The outcomes of these sessions revealed the participants' preference for an invite by mail through an address file, sent by a trusted organisation that provided a clear rationale for the free screening. The municipality was perceived as reliable, attributed to their role in providing housing for migrants. Therefore, the migrants were invited by letter with a clear reason for the invitation from the PHS - an organization clearly related to the municipality.’’.<br>As the Syrian migrants in the focus groups preferred this, we chose to commit to this sampling strategy.<br><br>If a comment is not addressed to your satisfaction, please let us know. Once more, we would like to thank you for your time, positive feedback, and valuable insights. We look forward to the rest of the process.<br><br>Thank you in advance.<br><br>Kind regards,<br><br>On behalf of all authors,<br>Chrissy Moonen
**Additional Information:**	
**Question**	**Response**
**Financial Disclosure**<br><br>Enter a financial disclosure statement that describes the sources of funding for the work included in this submission. Review the submission guidelines for detailed requirements. View published research articles from *PLOS ONE* for specific examples.<br><br>This statement is required for submission and **will appear in the published article** if the submission is accepted. Please make sure it is accurate.	Yes

Powered by Editorial Manager® and ProduXion Manager® from Aries Systems Corporation

**Funded studies**<br>Enter a statement with the following details:<br>• Initials of the authors who received each award<br>• Grant numbers awarded to each author<br>• The full name of each funder<br>• URL of each funder website<br>• Did the sponsors or funders play any role in the study design, data collection and analysis, decision to publish, or preparation of the manuscript?<br><br>Did you receive funding for this work?	
Please add funding details.<br>as follow-up to "**Financial Disclosure**<br><br>Enter a financial disclosure statement that describes the sources of funding for the work included in this submission. Review the submission guidelines for detailed requirements. View published research articles from *PLOS ONE* for specific examples.<br><br>This statement is required for submission and **will appear in the published article** if the submission is accepted. Please make sure it is accurate.<br><br>**Funded studies**<br>Enter a statement with the following details:<br>• Initials of the authors who received each award<br>• Grant numbers awarded to each author<br>• The full name of each funder<br>• URL of each funder website<br>• Did the sponsors or funders play any role in the study design, data collection and analysis, decision to publish, or preparation of the manuscript?<br><br>Did you receive funding for this work?"	This work was partly supported by the National Institute for Public Health and the Environment (https://www.rivm.nl/en). The funder had no role in study design, data collection and analysis, decision to publish, or preparation of the manuscript.
Please select the country of your main research funder (please select carefully as in some cases this is used in fee calculation).	NETHERLANDS - NL

as follow-up to "**Financial Disclosure**<br><br>Enter a financial disclosure statement that describes the sources of funding for the work included in this submission. Review the submission guidelines for detailed requirements. View published research articles from *PLOS ONE* for specific examples.<br><br>This statement is required for submission and **will appear in the published article** if the submission is accepted. Please make sure it is accurate.<br><br>**Funded studies**<br>Enter a statement with the following details:<br>• Initials of the authors who received each award<br>• Grant numbers awarded to each author<br>• The full name of each funder<br>• URL of each funder website<br>• Did the sponsors or funders play any role in the study design, data collection and analysis, decision to publish, or preparation of the manuscript?<br><br>Did you receive funding for this work?"	
**Competing Interests**<br><br>Use the instructions below to enter a competing interest statement for this submission. On behalf of all authors, disclose any competing interests that could be perceived to bias this work—acknowledging all financial support and any other relevant financial or non-financial competing interests.<br><br>This statement is required for submission and **will appear in the published article** if the submission is accepted. Please make sure it is accurate and that any funding sources listed in your Funding Information later in the submission form are also declared in your Financial Disclosure statement.	The authors have declared that no competing interests exist.

View published research articles from *PLOS ONE* for specific examples.<br><br>**NO authors have competing interests**<br><br>Enter: *The authors have declared that no competing interests exist.*<br><br>**Authors with competing interests**<br><br>Enter competing interest details beginning with this statement:<br><br>*I have read the journal's policy and the authors of this manuscript have the following competing interests: [insert competing interests here]*<br><br>* typeset	
**Ethics Statement**<br><br>Enter an ethics statement for this submission. This statement is required if the study involved:<br><br>• Human participants<br>• Human specimens or tissue<br>• Vertebrate animals or cephalopods<br>• Vertebrate embryos or tissues<br>• Field research<br><br>Write "N/A" if the submission does not require an ethics statement.<br><br>General guidance is provided below. Consult the submission guidelines for detailed instructions. **Make sure that all information entered here is included in the Methods section of the manuscript.**	The Medical Research Ethics Committees United (MEC-U) agreed that the research was outside the scope of the Dutch Medical Research Involving Human Subjects Act (WMO) (reference number W20.054). All participants provided written Informed Consent prior to participation.

Powered by Editorial Manager® and ProduXion Manager® from Aries Systems Corporation

**Format for specific study types**<br><br>**Human Subject Research (involving human participants and/or tissue)**<br>• Give the name of the institutional review board or ethics committee that approved the study<br>• Include the approval number and/or a statement indicating approval of this research<br>• Indicate the form of consent obtained (written/oral) or the reason that consent was not obtained (e.g. the data were analyzed anonymously)<br><br>**Animal Research (involving vertebrate animals, embryos or tissues)**<br>• Provide the name of the Institutional Animal Care and Use Committee (IACUC) or other relevant ethics board that reviewed the study protocol, and indicate whether they approved this research or granted a formal waiver of ethical approval<br>• Include an approval number if one was obtained<br>• If the study involved *non-human primates*, add *additional details* about animal welfare and steps taken to ameliorate suffering<br>• If anesthesia, euthanasia, or any kind of animal sacrifice is part of the study, include briefly which substances and/or methods were applied<br><br>**Field Research**<br><br>Include the following details if this study involves the collection of plant, animal, or other materials from a natural setting:<br>• Field permit number<br>• Name of the institution or relevant body that granted permission	
**Data Availability**<br><br>Authors are required to make all data underlying the findings described fully available, without restriction, and from the time of publication. PLOS allows rare exceptions to address legal and ethical concerns. See the PLOS Data Policy and FAQ for detailed information.	No - some restrictions will apply

A Data Availability Statement describing where the data can be found is required at submission. Your answers to this question constitute the Data Availability Statement and **will be published in the article**, if accepted.<br><br>**Important:** Stating 'data available on request from the author' is not sufficient. If your data are only available upon request, select 'No' for the first question and explain your exceptional situation in the text box.<br><br>Do the authors confirm that all data underlying the findings described in their manuscript are fully available without restriction?	
**Describe where the data may be found in full sentences. If you are copying our sample text, replace any instances of XXX with the appropriate details.**<br><br>• If the data are **held or will be held in a public repository**, include URLs, accession numbers or DOIs. If this information will only be available after acceptance, indicate this by ticking the box below. For example: *All XXX files are available from the XXX database (accession number(s) XXX, XXX.).*<br>• If the data are all contained **within the manuscript and/or Supporting Information files**, enter the following: *All relevant data are within the manuscript and its Supporting Information files.*<br>• If neither of these applies but you are able to provide **details of access elsewhere**, with or without limitations, please do so. For example:<br><br>*Data cannot be shared publicly because of [XXX]. Data are available from the XXX Institutional Data Access / Ethics Committee (contact via XXX) for researchers who meet the criteria for access to confidential data.*<br><br>*The data underlying the results presented in the study are available from (include the name of the third party*	The data of this study contain potentially identifying and sensitive participant information. Due to the General Data Protection Regulation, it is not allowed to distribute or share any personal data that can be traced back (direct or indirect) to an individual. In addition, publicly sharing the data would not be in accordance with the participants' consent obtained for this study. Therefore, data used and/or analysed during the study are available from the head of the data archiving of the Public Health Service South Limburg on reasonable request. Interested researchers should contact the head of the data-archiving of the Public Health Service South Limburg (Tamara Kleine: tamara.kleine@ggdzl.nl) when they would like to re-use data.

Powered by Editorial Manager® and ProduXion Manager® from Aries Systems Corporation

and contact information or URL).<br>• This text is appropriate if the data are owned by a third party and authors do not have permission to share the data.<br><br>* typeset	
Additional data availability information:	

Manuscript Click here to access/download;Manuscript;Manuscript.docx

Reaching Syrian migrants through Dutch municipal registries for hepatitis B and C point-of-care testing

Chrissy PB Moonen[1,2,¶*], Elfi EGH Brouwers[1,2], Christian JPA Hoebe[1,2,3], Nicole HTM Dukers-Muijrers[1,4], Jamila Bouchaara[1], Inge HM van Loo[3], Casper DJ den Heijer[1,2]

[1] Department of Sexual Health, Infectious Diseases and Environmental Health, Living Lab Public Health, South Limburg Public Health Service, Heerlen, The Netherlands

[2] Department of Social Medicine, Care and Public Health Research Institute (CAPHRI), Maastricht University, Maastricht, The Netherlands

[3] Department of Medical Microbiology, Infectious Diseases and Infection Prevention, Care and Public Health Research Institute (CAPHRI), Maastricht University Medical Center (MUMC+), Maastricht, The Netherlands

[4] Department of Health Promotion, Care and Public Health Research Institute (CAPHRI), Maastricht University, Maastricht, The Netherlands

*Corresponding author

E-mail: Chrissy.moonen@ggdzl.nl

Abstract

Undetected chronic hepatitis B virus (HBV) and hepatitis C virus (HCV) infections can lead to cirrhosis and liver cancer. Syrian migrants are the largest non-European migrant group in the Netherlands with HBV and HCV prevalence rates above 2%. This study aimed to reach Syrian migrants for HBV and HCV screening using point-of-care testing. A multifaceted strategy was employed to reach Syrian migrants aged ≥16 years from two Dutch municipalities for free-of-charge HBsAg and anti-HCV point-of-care testing (POCT) using finger prick blood at the regional Public Health Service. All were personally invited by the Public Health Service by postal mail, based on municipal registry data. Respondents' medical history data were analysed descriptively and data on age, sex, and municipality were compared with non-participating invitees, using Pearson's Chi-square test. Of the study population (N=832), 32.3% (n=269) attended the screening. The mean age of participants was 36 years (range 16-70), 59% were men, and 66.5% were unemployed. Non-participation was higher in the younger age groups (<30 years) ($p < .001$). The POCT using finger prick blood was well received. None tested HBsAg or anti-HCV positive. With approximately one-third participation, this study demonstrated successful reach of Syrian migrants for screening after a multifaceted strategy. However, while the reach could be considered successful, screening failed to demonstrate new infection in this key population. Thereby, other methods may be preferred to identify new HBV and HCV infections, such as opportunistic screening within existing care processes.

Introduction

Hepatitis is an inflammation of the liver that, if left untreated, can lead to liver cirrhosis and hepatocellular carcinoma (HCC) [1]. Hepatitis B virus (HBV) and hepatitis C virus (HCV) contributed to almost 98 per cent of all hepatitis-related deaths in 2017 [2]. An estimated 1.1 million deaths were attributable to these viruses in 2019 worldwide [3]. The World Health Organization (WHO) has called for action to eliminate hepatitis by the year 2030 [4]. Micro-elimination by screening high-risk groups is key to addressing hepatitis as a public health problem, especially given the often asymptomatic course of HBV and HCV [5]. Once the infection is identified, care can be initiated with treatment options that have greatly improved in recent years, particularly with the development of direct-acting antiviral (DAA) agents for HCV [6].

In response to the WHO call, the Dutch National Institute for Public Health and the Environment (RIVM) developed a National Hepatitis Plan in 2016 [7]. Major pillars in this plan are early infection detection and treatment, improved organization of hepatitis care, and national registration [7]. Despite the low incidence of acute HBV, chronic HBV, and acute HCV (0.6, 6, and 0.36 per 100.000 respectively) in the Netherlands [8], screening high-risk groups can further reduce the burden of hepatitis. In the Netherlands, the 25 regional Public Health Services (PHS) are responsible for public health, prevention, and health promotion at the municipal level.

Since first generation migrants account for most Dutch chronic HBV (81%) and HCV (60%) cases, targeted screening initiatives for this group should be explored [9]. In the Netherlands, Syrian migrants are the largest non-European risk group with HBV and HCV prevalence levels above 2%, indicating that Syrian migrants are a relevant target group for screening [10, 11]. Combined HBV and HCV screening of migrants in a low-endemic country such as the

Netherlands has been shown to be cost-effective [12]. However, high-risk groups such as migrants are often not well reached for screening and treatment [13].

This study is part of a nationally coordinated implementation research to reach first-generation non-European migrants for HBV and HCV screening, following the National Hepatitis Plan. In the present study, we aimed to 1) reach Syrian migrants in two Dutch municipalities by inviting them through municipal registries and 2) identify HBV and HCV infections in Syrian first-generation migrants by POCT using finger prick blood and to rapidly link infected individuals to care.

Materials and Methods

Study design and setting

In this cross-sectional study, Syrian migrants from two Dutch municipalities (Heerlen and Maastricht) were invited by the PHS of South Limburg for free rapid HBV and HCV POCT testing using finger prick blood at a local PHS site. The municipalities of Heerlen and Maastricht are home to the highest number of Syrian migrants in the region of South Limburg. The Medical Research Ethics Committees United (MEC-U) agreed that the research was outside the scope of the Dutch Medical Research Involving Human Subjects Act (WMO) (reference number W20.054).

Study population

The study population consisted of Syrian status holders living in the municipalities of Heerlen and Maastricht in the Netherlands. Only Syrian migrants (born in Syria) aged 16 years or older were invited for screening.

Multifaceted strategy

The strategy employed in this study was established by combining various elements, inspired by a comprehensive literature review and the insights gained through conducted focus groups prior to commencing the study (Fig 1) [14]. The elements are structured into several levels, namely the innovation level, the organisational level, the interaction level, and the migrant and community level. With the aim of eliminating barriers and facilitating participation in the screening, these elements were combined.

Fig 1. Components of our multifaceted strategy based on literature and focus groups

A Syrian physician employed by the PHS undertook focus groups with Syrian migrants, conducting separate sessions for men and women. The outcomes of these sessions revealed the participants' preference for an invite by mail through an address file, sent by a trusted organisation that provided a clear rationale for the free screening. The municipality was perceived as reliable, attributed to their role in providing housing for migrants. Therefore, the migrants were invited by letter with a clear reason for the invitation from the PHS - an organization clearly related to the municipality. Furthermore, the respondents preferred the screening at a conveniently located place, with options for evening screening. We complied to this by organising the screening at easily accessible PHS sites, reachable by public transportation and ensured screening opportunities during both afternoon and evening hours.

Besides facilitating factors, several barriers were pointed out in the focus groups, including the language gap and lack of awareness. At the interaction level, the language barrier was tackled by providing bilingual materials (in Dutch and Arabic) in B1 reading level. Additionally, translators and cultural mediators were present throughout the screening days, offering

linguistic and transcultural mediation, clarifying information, and answering questions. These roles were undertaken by native and cultural sensitive peers and care professionals. To address awareness, information was incorporated into the invitation letters, featured on the PHS website, and displayed on bilingual posters disseminated across locations regularly frequented by Syrians, including supermarkets, local mosques, community centres, grillrooms, and general practitioners' offices.

The systematic literature review conducted before this study endorsed addressing the language barrier, raising awareness, and ensuring an accessible screening process. In an effort to enhance visibility and knowledge, local mosques were engaged to encourage community participation in the screening. Moreover, the review highlighted POCT as a low-threshold innovation to potentially overcome screening barriers, facilitating prompt identification of infections and timely linkage to care for treatment initiation [15]. Certified, registered, and trained nurses administered the screening, with strict adherence to protocols to maintain a systematic and streamlined approach. Data management was conducted using standardized forms to ensure consistency and accuracy throughout the study. To limit the use of personal data and safeguard participant privacy, each invitee was assigned a unique participant code, which was used to link their tests to their respective identities.

Study protocol

Pre-screening

Name and address information were requested from the municipal registries to send invitation letters. Stakeholders, including general practitioners (GPs) in the municipality and the gastrointestinal-liver department of the regional hospitals, were informed of the study.

Subsequently, the study population received an invitation for voluntary, free, rapid testing for HBV and HCV.

Screening

The screening days took place on the 13th and the 15th of March 2023 in Heerlen and on the 8th and 10th of May 2023 in Maastricht. In setting these dates, the Islamic fasting month of Ramadan was considered. Non-participants received an invitation for a catch-up day at the 24th of April for Heerlen and the 24th of May for Maastricht.

Before screening, participants completed a written informed consent form with a unique participant code and a medical history questionnaire containing sociodemographic, behavioural and medical questions. Upon consent, the participant received a finger prick for the rapid tests. After screening, participants could wait in a waiting area, where drinks, treats and toys for children were provided. After twenty minutes, the test results were assessed and communicated by a PHS physician to the participant. Regardless of the test results, the participants' data were recorded in the medical records in accordance with legal requirements. In the case of a positive test result, a direct referral to care was embedded, including confirmation of the test result (serology via venepuncture), and when confirmed positive notification of the GP, referral to the hospital, and source and contact investigation.

Data collection and analysis

Pre-screening reason(s) for non-participation

Invitees who were unwilling or unable to participate could anonymously indicate their main reason(s) for non-participation through a QR code or link provided in the invite and reminder. This led to a dual-lingual (Dutch/Arabic) one-question online questionnaire into market

research software Crowdtech (ISO-20252 and ISO-27001 certified, London, UK). Twenty-three fixed answer options, which were inspired by the systematic literature review we conducted before this study, and one open response option were given [14]. After screening, data were extracted and analysed descriptively.

Questionnaire

Before screening, participants completed a medical history questionnaire on demographics, main reason for testing, hepatitis (vaccination) status and risk assessment. Participants could again choose a Dutch or an Arabic version. After the screening, the paper questionnaires were entered (translated for the Arabic version) into Crowdtech by the lead researcher (CM). After completion of the pilot study, data were extracted and ten percent of the data were randomly checked for errors by a management assistant. The education level was categorised according to the education system in Syria in not educated, practically educated (basic education certificate), combined educated (technical/vocational secondary education certificate, technical diploma certificate, certificate of associate degree/licensed assistant, and general secondary education certificate), or theoretically educated (academic bachelor or master). Employment status was categorised as working (part-time or full-time), non-working (unemployed, benefits, housewife/man, and retired) and student. Marital status was reduced to partner (married, unmarried partner) or no partner (no partner, widowed, divorced).

Point-of-care testing

A finger prick for a capillary blood sample was performed by a nurse for rapid HBV and HCV testing. The presence of hepatitis B surface antigen (HBsAg) was tested according to manufacturer’s protocol using DETERMINE™ HBsAg 2 by Abbott (Chicago, United States), which meets the requirements of the EU and WHO for analytical sensitivity. We chose this test

for its good clinical performance in validation studies [16, 17]. The WHO evaluated this test with a specificity and sensitivity of 100 (95% CI 98.8–100) and 100 (95% CI 98.2–100) respectively [18]. The test demonstrates user-friendliness with rapid results, and entails lower costs, requiring fewer trained professionals, infrastructure, and logistical resources compared to laboratory testing [16, 17, 19]. Having HCV antibodies (Anti-HCV) were tested according to manufacturer's protocol with the Food and Drug Administration (FDA) approved OraQuick® HCV Rapid Antibody Test by OraSure (Bethlehem, United States) [20]. As well as the Determine HBsAg 2, the OraQuick Anti-HCV test demonstrated good sensitivity and specificity in validation studies [20-24]. The WHO validation confirmed the test's specificity at 99.7% (95% CI 98.3–100.0%) and sensitivity at 100% (95% CI 97.8%–100.0%) [25]. Both tests hold the CE-certification, signifying their compliance with safety standards for sale and use in the European Economic Area, as evaluated by the Conformité Européenne (CE). Positive rapid test results are confirmed by the department of Medical Microbiology, Infection Prevention and Infectious Diseases of the Maastricht University Medical Centre (MUMC+) using HBsAg II and anti-HCV screening (Elecsys, Roche, Switzerland) on venous blood collected in Ethylenediaminetetraacetic acid (EDTA) blood tubes.

Comparison of participants with non-participants

Participants were compared with invited non-participants based on sex, age group, and municipality of administration of the test using the Pearson Chi-Square test (X^2) and multivariable analyses. The Statistical Package for the Social Sciences (SPSS, version 27.0, IBM, Armonk, USA) for Windows was used. A p value of 0.05 or less was considered as statistically significant.

Results

In total, 269 individuals were screened in Heerlen (n=156) and Maastricht (n=113) (Fig 2). Approximately five percent of the invitations were undeliverable. No HBsAg or anti-HCV was detected.

Fig 2. Flowchart of participation and test results

Study population and characteristics

The study population consisted of those who received the invitation and those eligible for screening, although they were not initially invited (N=832). Some individuals (n=23) were accidentally not invited, probably due to outdated information from the municipal registries, but accompanied relatives who were invited and were screened anyway. We also screened individuals outside the study population (n=10), as exclusion for care ~~was not congruent with the vision of the public health service~~. However, these individuals were not included in the analysis.

The majority of participants lacked Dutch language proficiency and therefore completed the Arabic questionnaire (72.1%) and needed translation during the screening and communication of the test results. Conversations with participants revealed that POCT using finger prick blood was well received, mostly because of the minimally invasive finger prick and short turnaround time of approximately 20 minutes for the test results. The nurses expressed their satisfaction with the ease of administering the point-of-care tests and appreciated the support provided by the cultural mediators.

The mean age of the participants was 35.7 years (range: 16-70 years) (Table 1). Most participants were men (59.1%, n=159) and non-working (66.5%, n=177). The majority of

participants had a partner (58.4%, n=150) and 47.5% (n=124) declared that they lived with a partner. On average, participants had lived in Syria for 26.9 years, and most mothers (99.6%, n=268) and fathers (96.3%, n=259) of the participants were born in Syria as well.

Table 1. Descriptive demographic characteristics

Characteristics, N=269[a]	n (%)
**Age in years**	
Mean (range), s	35.7 (16-70), 13.3
18-19	37 (13.8)
20-29	62 (23.0)
30-39	67 (24.9)
40-49	54 (20.1)
50-59	38 (14.1)
60+	11 (4.1)
**Sex**[b]	
Male	159 (59.1)
Female	110 (40.9)
**Level of education**[c]	
Uneducated	28 (10.5)
Practically educated	63 (23.6)
Combined educated	126 (47.2)
Theoretically educated	50 (18.7)
**Employment**[d] – multiple answers possible	
Working	75 (28.2)
Non-working	177 (66.5)
Student	60 (22.6)
**Relation status**[e]	
Partner	150 (58.4)
No partner	107 (41.6)
**Living situation** – multiple answers possible	
Alone	55 (21.1)
With partner	124 (47.5)
With children	81 (31.0)
With parents	63 (24.1)
**Years lived in Syria**	
Mean (range), s	26.9 (1-63), 14.4
**Country of birth mother**	
Syria	268 (99.6)
Other	1 (0.4)
**Country of birth father**	
Syria	259 (97.0)
Other	8 (3.0)

[a] Missing data in variables: level of education n=2 (0.7%), employment n=3 (1.1%), relation status n=12 (4.5%), living situation n=8 (3%), years lived in Syria n=13 (4.8%), and country of birth father n=2 (0.7%).

[b] Self-reported, no declared non-binary.

[c] Practically educated: basic education certificate. Combined educated: technical/vocational secondary education certificate, technical diploma certificate, certificate of associate degree/licensed assistant, or general secondary education certificate. Theoretically educated: academic bachelor or master.

[d] Working: working part-time or full-time. Non-working: unemployed, benefits, housewife/man, retired.
[e] Partner: married or unmarried partner. No partner: no partner, widowed, or divorced.

Previous HBV-vaccination and hepatitis status

Of the participants, 46.3% were unaware of their HBV vaccination status. A small proportion (15.3%) was certain about being vaccinated previously, and 38.4% were not vaccinated. Twelve participants (4.7%) indicated that they have (had) hepatitis; however, the majority of them (83.3%) did not know what type. However, past HCV infections were not considered, as these would have been detected through the anti-HCV screening. Of the (previous) infected individuals, three (25%) were treated.

Risk factors

Regarding risk factors for HBV and HCV, 25.3% (65/257) reported having undergone surgery abroad, 3.1% (8/257) indicated having donated an organ, and 5.1% (13/255) said they had used drugs intravenously. It is worth noting that the relatively high percentage claiming that they had used drugs intravenously may be explained by some participants' interpretation that drugs meant the same thing as medication. Therefore, the Arabic translation was revised following the second session. None (0/259) reported positivity for human immunodeficiency virus (HIV) or history of sexual contact with a person from the same sex.

Reasons to (not) get screened

The most common reason for attending screening was being invited (n=194). Other often-recurring reasons were 'I want certainty' (n=64) and 'I want to be sure I do not have it' (n=58).

Those who did not want to attend could indicate their main motive(s) anonymously online before screening. Twenty-three individuals declared their motives for not participating, and

most recurring reasons were not feeling sick/not having any symptoms (n=9), not having time (n=9), and already screened (n=4). Other indicated reasons were a lack of knowledge about hepatitis (n=3), lack of transportation (n=2), lack of risk perception (n=1), experienced language barrier (n=1), lack of faith in the PHS (n=1), being vaccinated (n=1), and afraid to get infected on site (n=1). Again, the vast majority of the respondents completed the non-response question in Arabic (91.3%).

Comparison of participants and non-participants

Participants and non-participants did not differ with regard to sex and municipality of residence, but did differ regarding age (Table 2). Non-participation was higher in the 16–19 and 20–29 age groups (Fig 3).

Table 2. Characteristics of the participants and non-participants

	Participants<br>n (% within participation)	Non-participants<br>n (% within participation)	P
**Sex**			.516
Male	159 (31.5)	346 (68.5)	
Female	110 (33.6)	217 (66.4)	
**Municipality**			.064
Heerlen	156 (35.1)	288 (64.9)	
Maastricht	113 (29.1)	275 (70.9)	
**Age group**			**<.001**
16-29 years	100 (23.4)	328 (76.6)	
30-70 years	169 (41.8)	235 (58.2)	

Fig 3. Bar chart of (non)participation per age group (N=832)

Discussion

In this study, we aimed to reach Syrian migrants for HBV and HCV screening via POCT using finger prick blood by a multifaceted strategy. Reach was considered successful as the participation rate was relatively high (32.3%). However, the usefulness of this strategy is still considered limited since HBV and HCV infection were not detected. HBV and HCV prevalence for both infections is suggested to be over 2% in Syrian migrants [10, 11] and the participants did report additional risk factors. However, 4.7% of participants reported having (had) hepatitis, indicating that already previously, the diagnosis was made, for example in Syria, e.g. during the mandatory premarital screening, or elsewhere in the Netherlands. Other possible explanations for the absence of present infections in the current screening include clearance of past (HBV) infections or potential infections, whether detected or undetected, among non-participants. Nevertheless, we lack certainty due to non-participation and lack of information about cleared HBV infections , as the HBV test did not provide information on this.

The multifaceted approach we have adopted appears to be a successful strategy to reach Syrian migrants for screening. This is supported by the turnup, as the one-third participation in this study is considerably higher than in studies with similar recruitment methods. For example, two Spanish studies in which the general Spanish population was invited by personal letter for HCV screening, showed participation rates of 11.2% and 4.1%, respectively [26, 27]. Compared to these Spanish studies, the participation rate was slightly higher in another Dutch project (17.8%), in which 10,000 migrants were invited per letter by the PHS [28]. The higher participation rate in the current study may be attributed to the multifaceted strategy, since this was developed based on a preceding extensive review on facilitators and barriers to screening [14]. This hypothesis is supported by the participation rate of 29.7% in another Dutch study

[29], in which additional efforts were made in addition to a personal invitation to recruit migrants from Afghanistan, Iran, Iraq, the former Soviet Republics, and Vietnam.

The ~~attendance to the screening~~ was higher among invitees over 30 years old than among younger invitees. Although age (distribution) is often underreported in prevalence studies, older age and male sex are associated with higher HBV prevalence rates [30, 31]. This is supported by a recent study in which Syrian blood donors in the age–18-25 years were less likely to be infected [32]. The screening method in the current approach reached more older individuals (over 30 years), who are more likely at a higher risk of infection. This rationale is confirmed by a Dutch study in which Syrian migrants younger than 25 years old were most often immune through vaccination and least often through infection [33].

In this study, POCT using finger prick blood was well received by both participants and staff and was preferred over venepuncture testing, aligning with previous findings [34]. Nursing staff found the tests easy to administer, which was echoed by the successful execution of all tests. Lebano, Hamed (35)Migrants face inequality due to more challenges in accessing healthcare compared to non-migrants, including communication barriers and discrimination (35). This makes the optimal use of a care moment even more valuable. POCT has the potential to improve the cascade of care and reduce loss to follow-up [35]. Moreover, the major demand for the Arabic questionnaire and the indispensability of a cultural mediator emphasize the importance of bilingual (informational) materials and (transcultural) translators during screening. We anticipate that the insights from this study hold considerable generalizability and can serve as an indication for other countries or regions adopting a similar approach for this population.

Limitations

Although name and address details were requested shortly before the invitations were sent out, the five percent undeliverable letters suggest that municipal records data are not sufficiently updated. Events that may have influenced screening participation included a regional transportation strike and elections for provincial councils and water boards (March 15, 2023) and a violent conflict in Maastricht between two groups of Syrian youths that made headlines (April 29, 2023). As noted by the Syrian (transcultural) translator involved in this pilot, the earthquake in Syria and Turkey in February 2023 caused significant emotional damage due to the loss of family and friends in the homeland. This and other concerns due to the earthquake may have also affected participation.

As for the pilot itself, implementation was labour-intensive, requiring careful planning of staff. Furthermore, screening was only offered to one migrant group in two of the sixteen municipalities in the South-Limburg region. The questionnaire was self-administered, making it prone to bias, for example because of the fear of stigmatization. Due to the absence of identified infections, we cannot draw conclusions regarding the risk factors for infection or evaluate follow-up care as we initially intended. Furthermore, the strategies have not been evaluated in an experimental setting, so the individual or combined effects are unknown. However, the design and lessons learned from this study can be valuable in setting up other screening projects to reach migrants for HBV and HCV screening.

Recommendations

For screening to best fit the target audience, we recommend exploring this with the target audience beforehand through, for example, focus groups. Considering the absence of infections in this study and that screening is very time-consuming, we recommend a more sustainable

approach by implementing HBV and HCV testing in existing care processes (opportunistic screening). However, the people reached during screening may not be those who are seen in regular care. Therefore, screening might still be a useful complementary action for reaching populations in the highest prevalence segment, for example >10% for HBV and >3% for HCV when looking at the global distribution maps of CDA Foundation's Polaris Observatory (11). As in most immigrant populations, language barriers were present, and hence, translated materials and translator/cultural mediators are essential when communicating with migrants. We also recommend greater diversity in the origins of staff in healthcare organizations to better serve individuals from other countries of birth. Furthermore, POCT can overcome miscommunication regarding the need for follow-up and reduce frequent loss to follow-up in target populations as multiple visits for diagnosis are unnecessary. Testing for multiple infectious diseases simultaneously by POCT can improve the cascade of care and may expedite the linkage to care [36]. To arrive at a good method to sustainably integrate HBV and HCV screening and meet the WHO call, more research is needed.

Conclusions

With about one-third participation rate, we can conclude that a multifaceted strategy for HBV and HCV screening, including an invite by letter is a good way to reach migrants for screening. Although no infections were detected, POCT using finger prick blood was well received and could yield benefits for hepatitis programs and patients. In populations in the highest prevalence segment, targeted screening by inviting migrant groups by letter may be appropriate. However, to adopt a more sustainable approach and to save personnel and time, we recommend exploring opportunistic screening in existing care processes.

Acknowledgements

Special thanks to the dedicated staff members who provided assistance before and during the screening period, and we extend extra gratitude to the translators for their work in providing translations. We want to express our gratefulness to the National Institute for Public Health and the Environment for funding this project. Furthermore, we would like to acknowledge the Medical Microbiology lab of Maastricht University Medical Centre+ for their expertise and their medical microbiological advice. We extend our appreciation to the Public Health Service of Groningen and Amsterdam for their willingness to share their thoughts and experiences during the implementation study. The authors acknowledge the research infrastructure provided by the Dutch Collaborative Academic practice for Public health Infectious diseases (CAPI). Lastly, the main author wants to thank the co-authors for their efforts and support.

References

1. Perz JF, Armstrong GL, Farrington LA, Hutin YJ, Bell BP. The contributions of hepatitis B virus and hepatitis C virus infections to cirrhosis and primary liver cancer worldwide. Journal of hepatology. 2006;45(4):529-38. doi: https://doi.org/10.1016/j.jhep.2006.05.013.
2. Liu Z, Shi O, Zhang T, Jin L, Chen X. Disease burden of viral hepatitis A, B, C and E: A systematic analysis. Journal of viral hepatitis. 2020;27(12):1284-96. doi: 10.1111/jvh.13371.
3. World Health Organization. Global progress report on HIV, viral hepatitis and sexually transmitted infections, 2021: accountability for the global health sector strategies 2016–2021: actions for impact: web annex 2: data methods. 2021.
4. World Health Organization. Global health sector strategy on viral hepatitis 2016-2021. Towards ending viral hepatitis. . Geneva: World Health Organization, 2016 June 2016. Report No.
5. World Health Organization. Guidelines on hepatitis B and C testing. February 2017. Geneva: World Health Organization, 2017 Contract No.: Licence: CC BY-NC-SA 3.0 IGO.
6. Cabibbo G, Celsa C, Calvaruso V, Petta S, Cacciola I, Cannavò MR, et al. Direct-acting antivirals after successful treatment of early hepatocellular carcinoma improve survival in HCV-cirrhotic patients. Journal of hepatology. 2019;71(2):265-73. doi: https://doi.org/10.1016/j.jhep.2019.03.027.
7. David SH, R., van Steenbergen J, Urbanus A. Meer dan opsporen : Nationaal hepatitisplan: een strategie voor actie. Bilthoven: Rijksinstituut voor Volksgezondheid en Milieu, 2016 1 November 2016. Report No.
8. Rijksinstituut voor Volksgezondheid en Milieu. Hepatitis B en C in Nederland, jaaroverzicht 2018 2019. Jaargang 2, Editie 1, 16 september 2019:[Available from: https://www.rivm.nl/sites/default/files/newsletters/101500-399-13567.html#anchor_name12.
9. Koopsen J, Van Steenbergen J, Richardus JH, Prins M, de Coul EO, Croes E, et al. Chronic hepatitis B and C infections in the Netherlands: estimated prevalence in risk groups and the general population. Epidemiology & Infection. 2019;147. Epub 14 March 2019. doi: https://doi.org/10.1017/S0950268819000359.
10. Schweitzer A, Horn J, Mikolajczyk RT, Krause G, Ott JJ. Estimations of worldwide prevalence of chronic hepatitis B virus infection: a systematic review of data published between 1965 and 2013. Lancet. 2015;386(10003):1546-55. Epub 20150728. doi: 10.1016/s0140-6736(15)61412-x. PubMed PMID: 26231459.
11. CDA Foundation’s Polaris Observatory. Global Distribution: hepatitis B and hepatitis C 2023 [updated 19 juli 202326 juli 2023]. Available from: https://cdafound.org/polaris-countries-distribution/.
12. Suijkerbuijk AW, Van Hoek AJ, Koopsen J, De Man RA, Mangen M-JJ, De Melker HE, et al. Cost-effectiveness of screening for chronic hepatitis B and C among migrant populations in a low endemic country. PloS one. 2018;13(11):e0207037. doi: https://doi.org/10.1371/journal.pone.0207037.
13. Cox AL, El-Sayed MH, Kao J-H, Lazarus JV, Lemoine M, Lok AS, et al. Progress towards elimination goals for viral hepatitis. Nature Reviews Gastroenterology & Hepatology. 2020;17(9):533-42. doi: https://doi.org/10.1038/s41575-020-0332-6.
14. Moonen CPB, den Heijer CDJ, Dukers-Muijrers NHTM, van Dreumel R, Steins SCJ, Hoebe CJPA. A systematic review of barriers and facilitators for hepatitis B and C screening among migrants in the EU/EEA region. Frontiers in Public Health. 2023;11. Epub 15 February 2023. doi: 10.3389/fpubh.2023.1118227.

15. Shenge JA, Osiowy C. Rapid diagnostics for hepatitis B and C viruses in low-and middle-income countries. Frontiers in Virology. 2021;1:742722. doi: https://doi.org/10.3389/fviro.2021.742722.
16. Avellon A, Ala A, Diaz A, Domingo D, Gonzalez R, Hidalgo L, et al. Clinical performance of Determine HBsAg 2 rapid test for Hepatitis B detection. Journal of Medical Virology. 2020;92(12):3403-11.
17. Ceesay A, Lemoine M, Cohen D, Chemin I, Ndow G. Clinical utility of the'Determine HBsAg'Point-of-Care Test for Diagnosis of Hepatitis B Surface Antigen in Africa. Expert Review of Molecular Diagnostics. 2022;22(5):497-505. doi: https://doi.org/10.1080/14737159.2022.2076595.
18. World Health Organization. WHO Prequalification of In Vitro Diagnostics: Public report. Product: Determine HBsAg 2. WHO, 2023 WHO reference number: PQDx 0451-013-00.
19. Xiao Y, Thompson AJ, Howell J. Point-of-care tests for hepatitis B: an overview. Cells. 2020;9(10):2233. doi: https://doi.org/10.3390/cells9102233.
20. Pallarés C, Carvalho-Gomes Â, Hontangas V, Conde I, Di Maira T, Aguilera V, et al. Performance of the OraQuick Hepatitis C virus antibody test in oral fluid and fingerstick blood before and after treatment-induced viral clearance. Journal of Clinical Virology. 2018;102:77-83.
21. Shivkumar S, Peeling R, Jafari Y, Joseph L, Pant Pai N. Accuracy of rapid and point-of-care screening tests for hepatitis C: a systematic review and meta-analysis. Annals of internal medicine. 2012;157(8):558-66. doi: 10.7326/0003-4819-157-8-201210160-00006.
22. Chevaliez S, Poiteau L, Rosa I, Soulier A, Roudot-Thoraval F, Laperche S, et al. Prospective assessment of rapid diagnostic tests for the detection of antibodies to hepatitis C virus, a tool for improving access to care. Clinical Microbiology and Infection. 2016;22(5):459. e1-. e6. doi: https://doi.org/10.1016/j.cmi.2016.01.009.
23. Khuroo MS, Khuroo NS, Khuroo MS. Diagnostic accuracy of point-of-care tests for hepatitis C virus infection: a systematic review and meta-analysis. PloS one. 2015;10(3):e0121450. doi: https://doi.org/10.1371/journal.pone.0121450.
24. Lee SR, Yearwood GD, Guillon GB, Kurtz LA, Fischl M, Friel T, et al. Evaluation of a rapid, point-of-care test device for the diagnosis of hepatitis C infection. J Clin Virol. 2010;48(1):15-7. Epub 20100401. doi: 10.1016/j.jcv.2010.02.018. PubMed PMID: 20362493.
25. World Health Organization. WHO Prequalification of In Vitro Diagnostics: Public Report. Product: OraQUick HCV Rapid Antibody Test Kit. WHO, 2017 WHO reference number: PQDx 0244-055-00.
26. Carvalho-Gomes ÂDc, Cubells ADc, Pallarés CDc, Hontangas VI, Conde IDc, Di Maira TI, et al. A population-based screening for hepatitis C antibodies and active infection using a point-of-care test in a low prevalence area. PLOS ONE [Internet]. 2020; 15(2).
27. Caballería L, Pera G, Bernad J, Canut S, Navarro E, Bruguera M. Strategies for the detection of hepatitis C viral infection in the general population. Revista clinica espanola. 2014;214(5):242-6. doi: 10.1016/j.rce.2014.01.024.
28. Niessen W, Benne R, van Zeijl J, van der Have J, Broer J. Is het schriftelijk oproepen van immigranten voor hepatitis B-onderzoek effectief? / IB 04-2013 2013. Jaargang 24, nummer 4:[Infectieziekten Bulletin]. Available from: https://www.rivm.nl/weblog/is-schriftelijk-oproepen-van-immigranten-voor-hepatitis-b-onderzoek-effectief-ib-04-2013.
29. Richter C, Ter Beest G, Gisolf E, Van Bentum P, Waegemaekers C, Swanink C, et al. Screening for chronic hepatitis B and C in migrants from Afghanistan, Iran, Iraq, the former Soviet Republics, and Vietnam in the Arnhem region, The Netherlands. Epidemiology & Infection. 2014;142(10):2140-6. doi: https://doi.org/10.1017/S0950268813003415.

30. Rossi C, Shrier I, Marshall L, Cnossen S, Schwartzman K, Klein MB, et al. Seroprevalence of chronic hepatitis B virus infection and prior immunity in immigrants and refugees: a systematic review and meta-analysis. 2012. doi: https://doi.org/10.1371/journal.pone.0044611.
31. Lee C, Emeto TI, Walsh N. Prevalence of hepatitis B virus amongst refugees, asylum seekers and internally displaced persons in low- and middle-income countries: A systematic review. Journal of viral hepatitis. 2023;30(1):4-18. doi: 10.1111/jvh.13770.
32. Alassad A, Al Rahwanji MJ, Yousfan A, Al Moualem S, Farhat A, Youssef LA. Seroprevalence and trends of hepatitis B virus, hepatitis C virus and human immunodeficiency virus in Syrian blood donors at Damascus University Blood Center between 2004 and 2021. Frontiers in Public Health. 2023;11. doi: 10.3389/fpubh.2023.1174638.
33. Freidl GS, Tostmann A, Curvers M, Ruijs WLM, Smits G, Schepp R, et al. Immunity against measles, mumps, rubella, varicella, diphtheria, tetanus, polio, hepatitis A and hepatitis B among adult asylum seekers in the Netherlands, 2016. Vaccine. 2018;36(12):1664-72. doi: 10.1016/j.vaccine.2018.01.079.
34. Duracinsky M, Thonon F, Bun S, Ben Nasr I, Dara AF, Lakhdari S, et al. Good acceptability of HIV, HBV, and HCV screening during immigration medical check-up amongst migrants in France in the STRADA study. PLoS One. 2020;15(6):e0235260. Epub 20200626. doi: 10.1371/journal.pone.0235260. PubMed PMID: 32589652; PubMed Central PMCID: PMCPMC7319329.
35. Lebano A, Hamed S, Bradby H, Gil-Salmerón A, Durá-Ferrandis E, Garcés-Ferrer J, et al. Migrants’ and refugees’ health status and healthcare in Europe: a scoping literature review. BMC Public Health. 2020;20(1):1039. doi: 10.1186/s12889-020-08749-8.
36. Bottero J, Boyd A, Gozlan J, Carrat F, Nau J, Pauti M-D, et al., editors. Simultaneous human immunodeficiency virus-hepatitis B-hepatitis C point-of-care tests improve outcomes in linkage-to-care: results of a randomized control trial in persons without healthcare coverage. Open forum infectious diseases; 2015: Oxford University Press.

List of abbreviations

HCC	Hepatocellular carcinoma
HBV	Hepatitis B virus
HCV	Hepatitis C virus
WHO	World Health Organization
DAA	Direct-acting antiviral agents
RIVM	The Dutch National Institute for Public Health and the Environment
PHS	Public Health Service
MEC-U	Medical Research Ethics Committees United
WMO	The Dutch Medical Research Involving Human Subjects Act
POCT	Point-of-care testing
GP	General practitioner

HBsAg	Hepatitis B surface antigen
Anti-HCV	Hepatitis C virus antibodies
FDA	Food and Drug Administration
CE	Conformité Européenne
MUMC+	Maastricht University Medical Centre
EDTA	Ethylenediaminetetraacetic acid
SPSS	The Statistical Package for the Social Sciences
HIV	Human immunodeficiency virus

Fig1

Click here to access/download;Figure;Fig1.tif

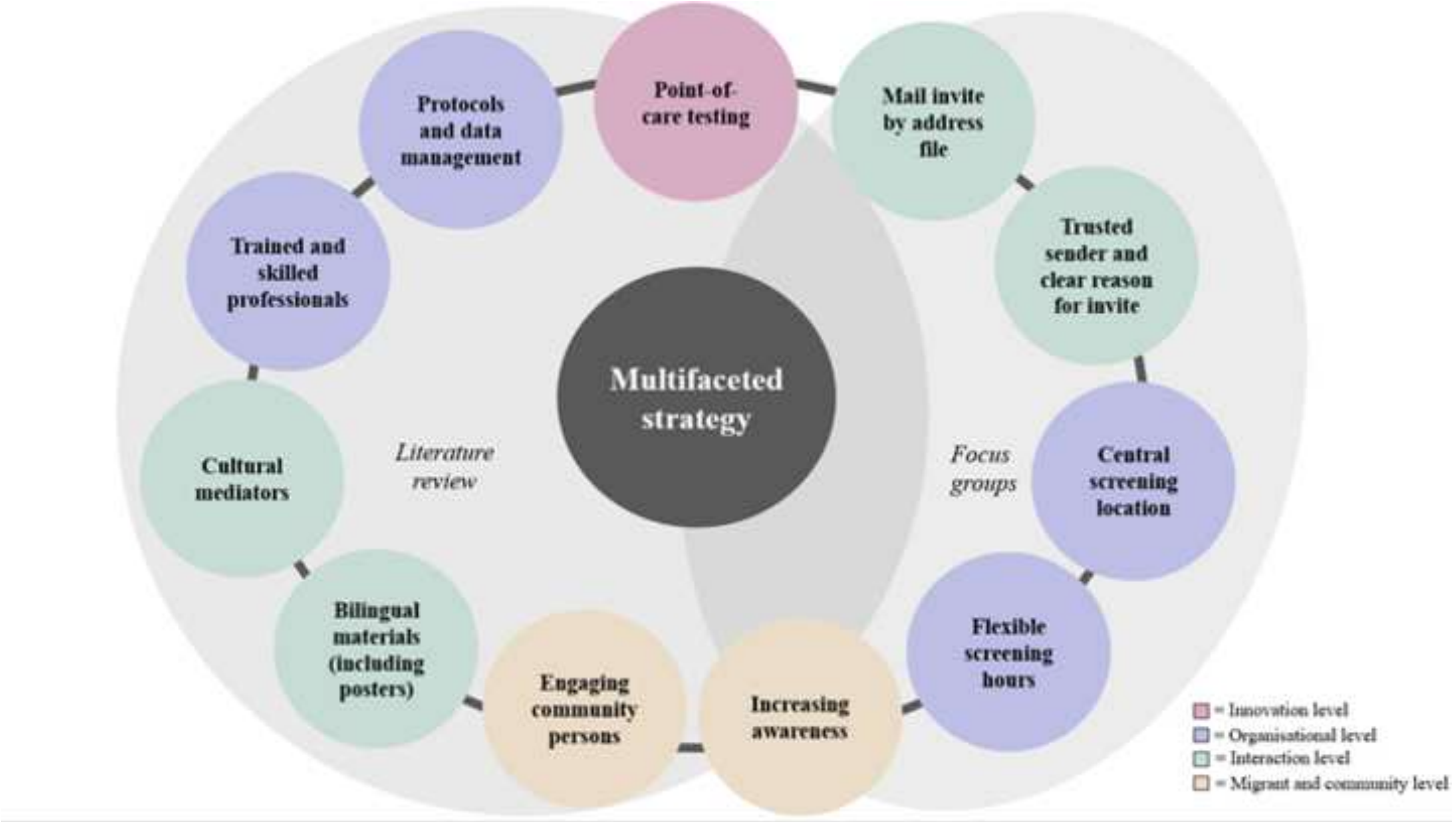

Fig2

Click here to access/download;Figure;Fig2.tif

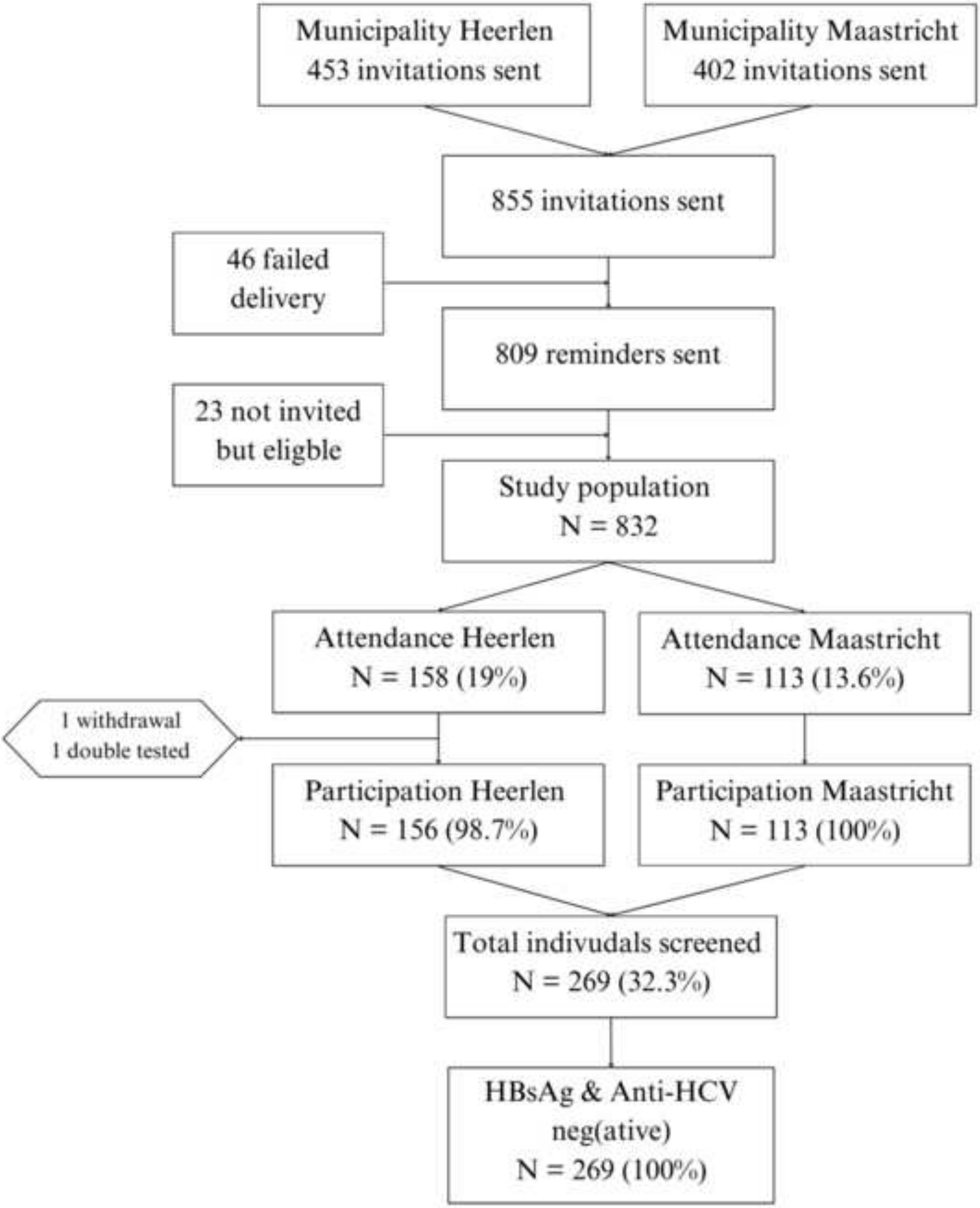

Fig3

Click here to access/download;Figure;Fig3.tif

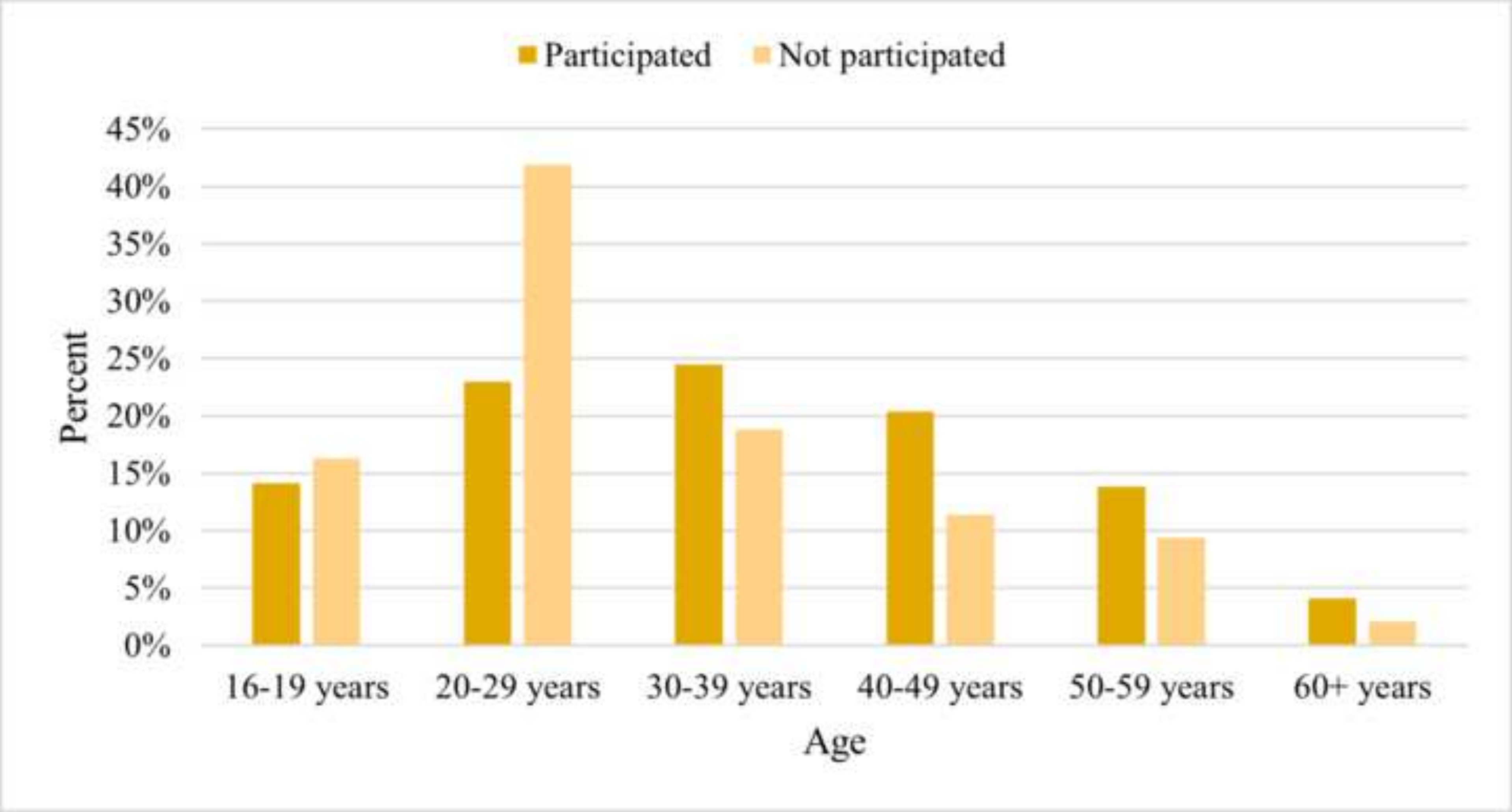

Reaching Syrian migrants through Dutch municipal registries for hepatitis B and C point-of-care testing

Chrissy PB Moonen[1,2,*], Elfi EGH Brouwers[1,2], Christian JPA Hoebe[1,2,3], Nicole HTM Dukers-Muijrers[1,4], Jamila Bouchaara[1], Inge HM van Loo[3], Casper DJ den Heijer[1,2]

[1] Department of Sexual Health, Infectious Diseases and Environmental Health, Living Lab Public Health, South Limburg Public Health Service, Heerlen, The Netherlands

[2] Department of Social Medicine, Care and Public Health Research Institute (CAPHRI), Maastricht University, Maastricht, The Netherlands

[3] Department of Medical Microbiology, Infectious Diseases and Infection Prevention, Care and Public Health Research Institute (CAPHRI), Maastricht University Medical Center (MUMC+), Maastricht, The Netherlands

[4] Department of Health Promotion, Care and Public Health Research Institute (CAPHRI), Maastricht University, Maastricht, The Netherlands

*Corresponding author

E-mail: Chrissy.moonen@ggdzl.nl

Abstract

Undetected chronic hepatitis B virus (HBV) and hepatitis C virus (HCV) infections can lead to cirrhosis and liver cancer. Syrian migrants are the largest non-European migrant group in the Netherlands with HBV and HCV prevalence rates above 2%. This study aimed to reach Syrian migrants for HBV and HCV screening using point-of-care testing. A multifaceted strategy was employed to reach Syrian migrants aged ≥16 years from two Dutch municipalities for free-of-charge HBsAg and anti-HCV point-of-care testing (POCT) using finger prick blood at the regional Public Health Service. All were personally invited by the Public Health Service by postal mail, based on municipal registry data. Respondents' medical history data were analysed descriptively and data on age, sex, and municipality were compared with non-participating invitees, using Pearson's Chi-square test. Of the study population (N=832), 32.3% (n=269) attended the screening. The mean age of participants was 36 years (range 16-70), 59% were men, and 66.5% were unemployed. Non-participation was higher in the younger age groups (<30 years) (p <.001). The POCT using finger prick blood was well received. None tested HBsAg or anti-HCV positive. With approximately one-third participation, this study demonstrated successful reach of Syrian migrants for screening after a multifaceted strategy. However, while the reach could be considered successful, screening failed to demonstrate new infection in this key population. Thereby, other methods may be preferred to identify new HBV and HCV infections, such as opportunistic screening within existing care processes.

~~**Keywords**~~

~~Hepatitis B, Hepatitis C, Point-of-Care Testing, Emigrants and Immigrants, Diagnostic Screening Programs~~

44

Introduction

Hepatitis is an inflammation of the liver that, if left untreated, can lead to liver cirrhosis and hepatocellular carcinoma (HCC) [1]. Hepatitis B virus (HBV) and hepatitis C virus (HCV) contributed to almost 98 per cent of all hepatitis-related deaths in 2017 [2]. An estimated 1.1 million deaths were attributable to these viruses in 2019 worldwide [3]. The World Health Organization (WHO) has called for action to eliminate hepatitis by the year 2030 [4]. Micro-elimination by screening high-risk groups is key to addressing hepatitis as a public health problem, especially given the often asymptomatic course of HBV and HCV [5]. Once the infection is identified, care can be initiated with treatment options that have greatly improved in recent years, particularly with the development of direct-acting antiviral (DAA) agents for HCV [6].

In response to the WHO call, the Dutch National Institute for Public Health and the Environment (RIVM) developed a National Hepatitis Plan in 2016 [7]. Major pillars in this plan are early infection detection and treatment, improved organization of hepatitis care, and national registration [7]. Despite the low incidence of acute HBV, chronic HBV, and acute HCV (0.6, 6, and 0.36 per 100.000 respectively) in the Netherlands [8], screening high-risk groups can further reduce the burden of hepatitis. In the Netherlands, the 25 regional Public Health Services (PHS) are responsible for public health, prevention, and health promotion at the municipal level.

Since first generation migrants account for most Dutch chronic HBV (81%) and HCV (60%) cases, targeted screening initiatives for this group should be explored [9]. In the Netherlands, Syrian migrants are the largest non-European risk group with HBV and HCV prevalence levels above 2%, indicating that Syrian migrants are a relevant target group for screening [10, 11]. Combined HBV and HCV screening of migrants in a low-endemic country such as the

Netherlands has been shown to be cost-effective [12]. However, high-risk groups such as migrants are often not well reached for screening and treatment [13].

This study is part of a nationally coordinated implementation research to reach first-generation non-European migrants for HBV and HCV screening, following the National Hepatitis Plan. In the present study, we aimed to 1) reach Syrian migrants in two Dutch municipalities by inviting them through municipal registries and 2) identify HBV and HCV infections in Syrian first-generation migrants by POCT using finger prick blood and to rapidly link infected individuals to care.

Materials and Methods

Study design and setting

In this cross-sectional study, Syrian migrants from two Dutch municipalities (Heerlen and Maastricht) were invited by the PHS of South Limburg for free rapid HBV and HCV POCT testing using finger prick blood at a local PHS site. The municipalities of Heerlen and Maastricht are home to the highest number of Syrian migrants in the region of South Limburg. The Medical Research Ethics Committees United (MEC-U) agreed that the research was outside the scope of the Dutch Medical Research Involving Human Subjects Act (WMO) (reference number W20.054).

Study population

The study population consisted of Syrian status holders living in the municipalities of Heerlen and Maastricht in the Netherlands. Only Syrian migrants (born in Syria) aged 16 years or older were invited for screening.

Multifaceted strategy

The strategy employed in this study was established by combining various elements, inspired by a comprehensive literature review and the insights gained through conducted focus groups prior to commencing the study (Fig 1) [14]. The elements are structured into several levels, namely the innovation level, the organisational level, the interaction level, and the migrant and community level. With the aim of eliminating barriers and facilitating participation in the screening, these elements were combined.

Fig 1. Components of our multifaceted strategy based on literature and focus groups

A Syrian physician employed by the PHS undertook focus groups with Syrian migrants, conducting separate sessions for men and women. The outcomes of these sessions revealed the participants' preference for an invite by mail through an address file, sent by a trusted organisation that provided a clear rationale for the free screening. The municipality was perceived as reliable, attributed to their role in providing housing for migrants. Therefore, the migrants were invited by letter with a clear reason for the invitation from the PHS - an organization clearly related to the municipality. Furthermore, the respondents preferred the screening at a conveniently located place, with options for evening screening. We complied to this by organising the screening at easily accessible PHS sites, reachable by public transportation and ensured screening opportunities during both afternoon and evening hours.

Besides facilitating factors, several barriers were pointed out in the focus groups, including the language gap and lack of awareness. At the interaction level, the language barrier was tackled by providing bilingual materials (in Dutch and Arabic) in B1 reading level. Additionally,

translators and cultural mediators were present throughout the screening days, offering linguistic and transcultural mediation, clarifying information, and answering questions. These roles were undertaken by native and cultural sensitive peers and care professionals. To address awareness, information was incorporated into the invitation letters, featured on the PHS website, and displayed on bilingual posters disseminated across locations regularly frequented by Syrians, including supermarkets, local mosques, community centres, grillrooms, and general practitioners' offices.

The systematic literature review conducted before this study endorsed addressing the language barrier, raising awareness, and ensuring an accessible screening process. In an effort to enhance visibility and knowledge, local mosques were engaged to encourage community participation in the screening. Moreover, the review highlighted ~~the point-of-care testing (POCT)~~POCT as a low-threshold innovation to potentially overcome screening barriers, facilitating prompt identification of infections and timely linkage to care for treatment initiation [15]. Certified, registered, and trained nurses administered the screening, with strict adherence to protocols to maintain a systematic and streamlined approach. Data management was conducted using standardized forms to ensure consistency and accuracy throughout the study. To limit the use of personal data and safeguard participant privacy, each invitee was assigned a unique participant code, which was used to link their tests to their respective identities.

Study protocol

Pre-screening

Name and address information were requested from the municipal registries to send invitation letters. Stakeholders, including general practitioners (GPs) in the municipality and the gastrointestinal-liver department of the regional hospitals, were informed of the study.

Subsequently, the study population received an invitation for voluntary, free, rapid testing for HBV and HCV.

Screening

The screening days took place on the 13th and the 15th of March 2023 in Heerlen and on the 8th and 10th of May 2023 in Maastricht. In setting these dates, the Islamic fasting month of Ramadan was considered. Non-participants received an invitation for a catch-up day at the 24th of April for Heerlen and the 24th of May for Maastricht.

Before screening, participants completed a written informed consent form with a unique participant code and a medical history questionnaire containing sociodemographic, behavioural and medical questions. Upon consent, the participant received a finger prick for the rapid tests. After screening, participants could wait in a waiting area, where drinks, treats and toys for children were provided. After twenty minutes, the test results were assessed and communicated by a PHS physician to the participant. Regardless of the test results, the participants' data were recorded in the medical records in accordance with legal requirements. In the case of a positive test result, a direct referral to care was embedded, including confirmation of the test result (serology via venepuncture), and when confirmed positive notification of the GP, referral to the hospital, and source and contact investigation.

Data collection and analysis

Pre-screening reason(s) for non-participation

Invitees who were unwilling or unable to participate could anonymously indicate their main reason(s) for non-participation through a QR code or link provided in the invite and reminder. This led to a dual-lingual (Dutch/Arabic) one-question online questionnaire into market

research software Crowdtech (ISO-20252 and ISO-27001 certified, London, UK). Twenty-three fixed answer options, which were inspired by the systematic literature review we conducted before this study, and one open response option were given [14]. After screening, data were extracted and analysed descriptively.

Questionnaire

Before screening, participants completed a medical history questionnaire on demographics, main reason for testing, hepatitis (vaccination) status and risk assessment. Participants could again choose a Dutch or an Arabic version. After the screening, the paper questionnaires were entered (translated for the Arabic version) into Crowdtech by the lead researcher (CM). After completion of the pilot study, data were extracted and ten percent of the data were randomly checked for errors by a management assistant. The education level was categorised according to the education system in Syria in not educated, practically educated (basic education certificate), combined educated (technical/vocational secondary education certificate, technical diploma certificate, certificate of associate degree/licensed assistant, and general secondary education certificate), or theoretically educated (academic bachelor or master). Employment status was categorised as working (part-time or full-time), non-working (unemployed, benefits, housewife/man, and retired) and student. Marital status was reduced to partner (married, unmarried partner) or no partner (no partner, widowed, divorced).

Point-of-care testing

A finger prick for a capillary blood sample was performed by a nurse for rapid HBV and HCV testing. The presence of hepatitis B surface antigen (HBsAg) was tested according to manufacturer’s protocol using DETERMINE™ HBsAg 2 by Abbott (Chicago, United States), which meets the requirements of the EU and WHO for analytical sensitivity. We chose this test

for its good clinical performance in validation studies [16, 17]. The WHO evaluated this test with a specificity and sensitivity of 100 (95% CI 98.8–100) and 100 (95% CI 98.2–100) respectively [18]. The test demonstrates user-friendliness with rapid results, and entails lower costs, requiring fewer trained professionals, infrastructure, and logistical resources compared to laboratory testing [16, 17, 19]. Having HCV antibodies (Anti-HCV) were tested according to manufacturer's protocol with the Food and Drug Administration (FDA) approved OraQuick® HCV Rapid Antibody Test by OraSure (Bethlehem, United States) [20]. As well as the Determine HBsAg 2, the OraQuick Anti-HCV test demonstrated good sensitivity and specificity in validation studies [20-24]. The WHO validation confirmed the test's specificity at 99.7% (95% CI 98.3–100.0%) and sensitivity at 100% (95% CI 97.8%–100.0%) [25]. Both tests hold the CE-certification, signifying their compliance with safety standards for sale and use in the European Economic Area, as evaluated by the Conformité Européenne (CE). Positive rapid test results are confirmed by the department of Medical Microbiology, Infection Prevention and Infectious Diseases of the Maastricht University Medical Centre (MUMC+) using HBsAg II and anti-HCV screening (Elecsys, Roche, Switzerland) on venous blood collected in Ethylenediaminetetraacetic acid (EDTA) blood tubes.

Comparison of participants with non-participants

Participants were compared with invited non-participants based on sex, age group, and municipality of administration of the test using the Pearson Chi-Square test (X^2) and multivariable analyses. The Statistical Package for the Social Sciences (SPSS, version 27.0, IBM, Armonk, USA) for Windows was used. A p value of 0.05 or less was considered as statistically significant.

Results

In total, 269 individuals were screened in Heerlen (n=156) and Maastricht (n=113) (Fig 2). Approximately five percent of the invitations were undeliverable. No HBsAg or anti-HCV was detected.

Fig 2. Flowchart of participation and test results

Formatted: Font: 10 pt

Study population and characteristics

The study population consisted of those who received the invitation and those eligible for screening, although they were not initially invited (N=832). Some individuals (n=23) were accidentally not invited, probably due to outdated information from the municipal registries, but accompanied relatives who were invited and were screened anyway. We also screened individuals outside the study population (n=10), as exclusion for care was not congruent with the vision of the public health service. However, these individuals were not included in the analysis.

The majority of participants lacked Dutch language proficiency and therefore completed the Arabic questionnaire (72.1%) and needed translation during the screening and communication of the test results. Conversations with participants revealed that POCT using finger prick blood was well received, mostly because of the minimally invasive finger prick and short turnaround time of approximately 20 minutes for the test results. The nurses expressed their satisfaction with the ease of administering the point-of-care tests and appreciated the support provided by the cultural mediators.

The mean age of the participants was 35.7 years (range: 16-70 years) (Table 1). Most participants were men (59.1%, n=159) and non-working (66.5%, n=177). The majority of

participants had a partner (58.4%, n=150) and 47.5% (n=124) declared that they lived with a partner. On average, participants had lived in Syria for 26.9 years, and most mothers (99.6%, n=268) and fathers (96.3%, n=259) of the participants were born in Syria as well.

Table 1. Descriptive demographic characteristics

Formatted: Font: 10 pt

Characteristics, N=269[a]	n (%)
**Age in years**	
Mean (range), s	35.7 (16-70), 13.3
18-19	37 (13.8)
20-29	62 (23.0)
30-39	67 (24.9)
40-49	54 (20.1)
50-59	38 (14.1)
60+	11 (4.1)
**Sex[b]**	
Male	159 (59.1)
Female	110 (40.9)
**Level of education[c]**	
Uneducated	28 (10.5)
Practically educated	63 (23.6)
Combined educated	126 (47.2)
Theoretically educated	50 (18.7)
**Employment[d]** – multiple answers possible	
Working	75 (28.2)
Non-working	177 (66.5)
Student	60 (22.6)
**Relation status[e]**	
Partner	150 (58.4)
No partner	107 (41.6)
**Living situation** – multiple answers possible	
Alone	55 (21.1)
With partner	124 (47.5)
With children	81 (31.0)
With parents	63 (24.1)
**Years lived in Syria**	
Mean (range), s	26.9 (1-63), 14.4
**Country of birth mother**	
Syria	268 (99.6)
Other	1 (0.4)
**Country of birth father**	
Syria	259 (97.0)
Other	8 (3.0)
**Characteristics, N=269[a]**	n (%)
**Age in years**	
Mean (range), s	35.7 (16-70), 13.3
18-19	37 (13.8)
20-29	62 (23.0)

30-39	67 (24.9)
40-49	54 (20.1)
50-59	38 (14.1)
60+	11 (4.1)
**Sex**[b]	
Male	159 (59.1)
Female	110 (40.9)
**Level of education**[c]	
Uneducated	28 (10.5)
Practically educated	63 (23.6)
Combined educated	126 (47.2)
Theoretically educated	50 (18.7)
**Employment**[d] – multiple answers possible	
Working	75 (28.2)
Non-working	177 (66.5)
Student	60 (22.6)
**Relation status**[e]	
Partner	150 (58.4)
No partner	107 (41.6)
**Living situation** – multiple answers possible	
Alone	55 (21.1)
With partner	124 (47.5)
With children	81 (31.0)
With parents	63 (24.1)
**Years lived in Syria**	
Mean (range), s	26.9 (1-63), 14.4
**Country of birth mother**	
Syria	268 (99.6)
Other	1 (0.4)
**Country of birth father**	
Syria	259 (97.0)
Other	8 (3.0)

[a] Missing data in variables: level of education n=2 (0.7%), employment n=3 (1.1%), relation status n=12 (4.5%), living situation n=8 (3%), years lived in Syria n=13 (4.8%), and country of birth father n=2 (0.7%).

[b] Self-reported, no declared non-binary.

[c] Practically educated: basic education certificate. Combined educated: technical/vocational secondary education certificate, technical diploma certificate, certificate of associate degree/licensed assistant, or general secondary education certificate. Theoretically educated: academic bachelor or master.

[d] Working: working part-time or full-time. Non-working: unemployed, benefits, housewife/man, retired.

[e] Partner: married or unmarried partner. No partner: no partner, widowed, or divorced.

Previous HBV-vaccination and hepatitis status

Of the participants, 46.3% were unaware of their HBV vaccination status. A small proportion (15.3%) was certain about being vaccinated previously, and 38.4% were not vaccinated. Twelve participants (4.7%) indicated that they have (had) hepatitis; however, the majority of them

(83.3%) did not know what type. However, past HCV infections were not considered, as these would have been detected through the anti-HCV screening. Of the (previous) infected individuals, three (25%) were treated.

Risk factors

Regarding risk factors for HBV and HCV, 25.3% (65/257) reported having undergone surgery abroad, 3.1% (8/257) indicated having donated an organ, and 5.1% (13/255) said they had used drugs intravenously. It is worth noting that the relatively high percentage claiming that they had used drugs intravenously may be explained by some participants' interpretation that drugs meant the same thing as medication. Therefore, the Arabic translation was revised following the second session. None (0/259) reported positivity for human immunodeficiency virus (HIV) or history of sexual contact with a person from the same sex.

Reasons to (not) get screened

The most common reason for attending screening was being invited (n=194). Other often-recurring reasons were 'I want certainty' (n=64) and 'I want to be sure I do not have it' (n=58).

Those who did not want to attend could indicate their main motive(s) anonymously online before screening. Twenty-three individuals declared their motives for not participating, and most recurring reasons were not feeling sick/not having any symptoms (n=9), not having time (n=9), and already screened (n=4).~~)~~ Other indicated reasons were a lack of knowledge about hepatitis (n=3), lack of transportation (n=2), lack of risk perception (n=1), experienced language barrier (n=1), lack of faith in the PHS (n=1), being vaccinated (n=1), and afraid to get infected on site (n=1). Again, the vast majority of the respondents completed the non-response question in Arabic (91.3%).

Comparison of participants and non-participants

Participants and non-participants did not differ with regard to sex and municipality of residence, but did differ regarding age (Table 2). Non-participation was higher in the 16–19 and 20–29 age groups (Fig 3).

Table 2. Characteristics of the participants and non-participants

	Participants n (% within participation)	Non-participants n (% within participation)	P
**Sex**			.516
Male	159 (31.5)	346 (68.5)	
Female	110 (33.6)	217 (66.4)	
**Municipality**			.064
Heerlen	156 (35.1)	288 (64.9)	
Maastricht	113 (29.1)	275 (70.9)	
**Age group**			**<.001**
16-29 years	100 (23.4)	328 (76.6)	
30-70 years	169 (41.8)	235 (58.2)	

Fig 3. Bar chart of ~~part~~(non)participation per age group (N=832)

Formatted: Font: 10 pt

Formatted Table

Formatted: Font: 10 pt

Discussion

In this study, we aimed to reach Syrian migrants for HBV and HCV screening via POCT using finger prick blood by a multifaceted strategy. Reach was considered successful as the participation rate was relatively high (32.3%). However, the usefulness of this strategy is still considered limited since HBV and HCV infection were not detected. HBV and HCV prevalence for both infections is suggested to be over 2% in Syrian migrants [10, 11] and the participants did report additional risk factors. However, 4.7% of participants reported having (had) hepatitis, indicating that already previously, the diagnosis was made, ~~. F~~for example in Syria, e.g. during the mandatory premarital screening, or elsewhere in the Netherlands. Other possible explanations for the absence of present infections in the current screening~~,~~ include clearance of past (HBV) infections or potential infections, whether detected or undetected, among non-participant~~tor~~s. Nevertheless, we lack certainty due to non-participation and lack of information about ~~their~~ cleared HBV infections ~~hepatitis status.~~, as the HBV test did not provide information on this.

The multifaceted approach we have adopted appears to be a successful strategy to reach Syrian migrants for screening. This is supported by the turnup, as the one-third participation in this study is considerably higher than in studies with similar recruitment methods. For example, two Spanish ~~the~~ studies ~~by~~ [26] ~~and~~ [27]~~,~~ in which the general Spanish population was invited by personal letter for HCV screening, showed participation rates of 11.2% and 4.1%, respectively [26, 27]. Compared to these Spanish studies, the participation rate was slightly higher in another Dutch project (17.8%), in which 10,000 migrants were invited per letter by the PHS [28]. The higher participation rate in the current study may be attributed to the multifaceted strategy, since this was developed based on a preceding extensive review on facilitators and barriers to screening [14]. This hypothesis is supported by the participation rate of 29.7% in another Dutch

study [29], in which additional efforts were made in addition to a personal invitation to recruit migrants from Afghanistan, Iran, Iraq, the former Soviet Republics, and Vietnam.

The attendance to the screening was higher among invitees over 30 years old than among younger invitees. Although age (distribution) is often underreported in prevalence studies, older age and male sex are associated with higher HBV prevalence rates [30, 31]. This is supported by a recent study by [32] in which Syrian blood donors in the age–18-25 years were less likely to be infected [32]. –The screening method in the current approach reached more older individuals (over 30 years), whom are more likely at a higher risk of infection. This rationale is confirmed by a Dutch study in which Syrian migrants younger than 25 years old were most often immune through vaccination and least often through infection [33].

In this study, POCT using finger prick blood was well received by both participants and staff and was preferred over venepuncture testing, aligning with the previous findings of [34]. Nursing staff found the tests easy to administer, which was echoed by the successfully execution of all tests.

As highlighted by Lebano, Hamed (35)M, migrants face inequality due to more challenges in accessing healthcare compared to non-migrants, including communication barriers and discrimination (35). This makes the optimal use of a care moment even more valuable. POCT has the potential to improve the cascade of care and reduce loss to follow-up [35]. Moreover, the major demand for the Arabic questionnaire and the indispensability of a cultural mediator emphasize the importance of bilingual (informational) materials and (transcultural) translators during screening. We anticipate that the insights from this study hold considerable generalizability and can serve as an indication for other countries or regions adopting a similar approach for this population.

Limitations

Although name and address details were requested shortly before the invitations were sent out, the five percent undeliverable letters suggest that municipal records data are not sufficiently updated. Events that may have influenced screening participation included a regional transportation strike and elections for provincial councils and water boards (March 15, 2023) and a violent conflict in Maastricht between two groups of Syrian youths that made headlines (April 29, 2023). As noted by the Syrian (transcultural) translator involved in this pilot, the earthquake in Syria and Turkey in February 2023 caused significant emotional damage due to the loss of family and friends in the homeland. This and other concerns due to the earthquake may have also affected participation.

As for the pilot itself, implementation was labour-intensive, requiring careful planning of staff. Furthermore, screening was only offered to one migrant group in two of the sixteen municipalities in the South-Limburg region. The questionnaire was self-administered, making it prone to bias, for example because of the fear of stigmatization. Due to the absence of identified infections, we cannot draw conclusions regarding the risk factors for infection or evaluate follow-up care as we initially intended. Furthermore, the strategies have not been evaluated in an experimental setting, so the individual or combined effects are unknown. However, the design and lessons learned from this study can be valuable in setting up other screening projects to reach migrants for HBV and HCV screening.

Recommendations

For screening to best fit the target audience, we recommend exploring this with the target audience beforehand through, for example, focus groups. Considering the absence of infections in this study and that screening is very time-consuming, we recommend a more sustainable

approach by implementing HBV and HCV testing in existing care processes (opportunistic screening). However, the people reached during screening may not be those who are seen in regular care. Therefore, screening might still be a useful complementary action for reaching populations in the highest prevalence segment, for example >10% for HBV and >3% for HCV when looking at the global distribution maps of CDA Foundation's Polaris Observatory (11). As in most immigrant populations, language barriers were present, and hence, translated materials and translator/cultural mediators are essential when communicating with migrants. We also recommend greater diversity in the origins of staff in healthcare organizations to better serve individuals from other countries of birth. Furthermore, POCT can overcome miscommunication regarding the need for follow-up and reduce frequent loss to follow-up in target populations as multiple visits for diagnosis are unnecessary. Testing for multiple infectious diseases simultaneously by POCT can improve the cascade of care and may expedite the linkage to care [36]. To arrive at a good method to sustainably integrate HBV and HCV screening and meet the WHO call, more research is needed.

Conclusions

With about one-third participation rate, we can conclude that a multifaceted strategy for HBV and HCV screening, including an invite by letter is a good way to reach migrants for screening. Although no infections were detected, POCT using finger prick blood was well received and could yield benefits for hepatitis programs and patients. In populations in the highest prevalence segment, targeted screening by inviting migrant groups by letter may be appropriate. However, to adopt a more sustainable approach and to save personnel and time, we recommend exploring opportunistic screening in existing care processes.

Acknowledgements

Special thanks to the dedicated staff members who provided assistance before and during the screening period, and we extend extra gratitude to the translators for their work in providing translations. We want to express our gratefulness to the National Institute for Public Health and the Environment for funding this project. Furthermore, we would like to acknowledge the Medical Microbiology lab of Maastricht University Medical Centre+ for their expertise and their medical microbiological advice. We extend our appreciation to the Public Health Service of Groningen and Amsterdam for their willingness to share their thoughts and experiences during the implementation study. The authors acknowledge the research infrastructure provided by the Dutch Collaborative Academic practice for Public health Infectious diseases (CAPI). Lastly, the main author wants to thank the co-authors for their efforts and support.

Formatted: Font: 14 pt

References

1. Perz JF, Armstrong GL, Farrington LA, Hutin YJ, Bell BP. The contributions of hepatitis B virus and hepatitis C virus infections to cirrhosis and primary liver cancer worldwide. Journal of hepatology. 2006;45(4):529-38. doi: https://doi.org/10.1016/j.jhep.2006.05.013.
2. Liu Z, Shi O, Zhang T, Jin L, Chen X. Disease burden of viral hepatitis A, B, C and E: A systematic analysis. Journal of viral hepatitis. 2020;27(12):1284-96. doi: 10.1111/jvh.13371.
3. World Health Organization. Global progress report on HIV, viral hepatitis and sexually transmitted infections, 2021: accountability for the global health sector strategies 2016–2021: actions for impact: web annex 2: data methods. 2021.
4. World Health Organization. Global health sector strategy on viral hepatitis 2016-2021. Towards ending viral hepatitis. . Geneva: World Health Organization, 2016 June 2016. Report No.
5. World Health Organization. Guidelines on hepatitis B and C testing. February 2017. Geneva: World Health Organization, 2017 Contract No.: Licence: CC BY-NC-SA 3.0 IGO.
6. Cabibbo G, Celsa C, Calvaruso V, Petta S, Cacciola I, Cannavò MR, et al. Direct-acting antivirals after successful treatment of early hepatocellular carcinoma improve survival in HCV-cirrhotic patients. Journal of hepatology. 2019;71(2):265-73. doi: https://doi.org/10.1016/j.jhep.2019.03.027.
7. David SH, R., van Steenbergen J, Urbanus A. Meer dan opsporen : Nationaal hepatitisplan: een strategie voor actie. Bilthoven: Rijksinstituut voor Volksgezondheid en Milieu, 2016 1 November 2016. Report No.
8. Rijksinstituut voor Volksgezondheid en Milieu. Hepatitis B en C in Nederland, jaaroverzicht 2018 2019. Jaargang 2, Editie 1, 16 september 2019:[Available from: https://www.rivm.nl/sites/default/files/newsletters/101500-399-13567.html#anchor_name12.
9. Koopsen J, Van Steenbergen J, Richardus JH, Prins M, de Coul EO, Croes E, et al. Chronic hepatitis B and C infections in the Netherlands: estimated prevalence in risk groups and the general population. Epidemiology & Infection. 2019;147. Epub 14 March 2019. doi: https://doi.org/10.1017/S0950268819000359.
10. Schweitzer A, Horn J, Mikolajczyk RT, Krause G, Ott JJ. Estimations of worldwide prevalence of chronic hepatitis B virus infection: a systematic review of data published between 1965 and 2013. Lancet. 2015;386(10003):1546-55. Epub 20150728. doi: 10.1016/s0140-6736(15)61412-x. PubMed PMID: 26231459.
11. CDA Foundation's Polaris Observatory. Global Distribution: hepatitis B and hepatitis C 2023 [updated 19 juli 202326 juli 2023]. Available from: https://cdafound.org/polaris-countries-distribution/.
12. Suijkerbuijk AW, Van Hoek AJ, Koopsen J, De Man RA, Mangen M-JJ, De Melker HE, et al. Cost-effectiveness of screening for chronic hepatitis B and C among migrant populations in a low endemic country. PloS one. 2018;13(11):e0207037. doi: https://doi.org/10.1371/journal.pone.0207037.
13. Cox AL, El-Sayed MH, Kao J-H, Lazarus JV, Lemoine M, Lok AS, et al. Progress towards elimination goals for viral hepatitis. Nature Reviews Gastroenterology & Hepatology. 2020;17(9):533-42. doi: https://doi.org/10.1038/s41575-020-0332-6.
14. Moonen CPB, den Heijer CDJ, Dukers-Muijrers NHTM, van Dreumel R, Steins SCJ, Hoebe CJPA. A systematic review of barriers and facilitators for hepatitis B and C screening among migrants in the EU/EEA region. Frontiers in Public Health. 2023;11. Epub 15 February 2023. doi: 10.3389/fpubh.2023.1118227.

15. Shenge JA, Osiowy C. Rapid diagnostics for hepatitis B and C viruses in low-and middle-income countries. Frontiers in Virology. 2021;1:742722. doi: https://doi.org/10.3389/fviro.2021.742722.
16. Avellon A, Ala A, Diaz A, Domingo D, Gonzalez R, Hidalgo L, et al. Clinical performance of Determine HBsAg 2 rapid test for Hepatitis B detection. Journal of Medical Virology. 2020;92(12):3403-11.
17. Ceesay A, Lemoine M, Cohen D, Chemin I, Ndow G. Clinical utility of the'Determine HBsAg'Point-of-Care Test for Diagnosis of Hepatitis B Surface Antigen in Africa. Expert Review of Molecular Diagnostics. 2022;22(5):497-505. doi: https://doi.org/10.1080/14737159.2022.2076595.
18. World Health Organization. WHO Prequalification of In Vitro Diagnostics: Public report. Product: Determine HBsAg 2. WHO, 2023 WHO reference number: PQDx 0451-013-00.
19. Xiao Y, Thompson AJ, Howell J. Point-of-care tests for hepatitis B: an overview. Cells. 2020;9(10):2233. doi: https://doi.org/10.3390/cells9102233.
20. Pallarés C, Carvalho-Gomes Â, Hontangas V, Conde I, Di Maira T, Aguilera V, et al. Performance of the OraQuick Hepatitis C virus antibody test in oral fluid and fingerstick blood before and after treatment-induced viral clearance. Journal of Clinical Virology. 2018;102:77-83.
21. Shivkumar S, Peeling R, Jafari Y, Joseph L, Pant Pai N. Accuracy of rapid and point-of-care screening tests for hepatitis C: a systematic review and meta-analysis. Annals of internal medicine. 2012;157(8):558-66. doi: 10.7326/0003-4819-157-8-201210160-00006.
22. Chevaliez S, Poiteau L, Rosa I, Soulier A, Roudot-Thoraval F, Laperche S, et al. Prospective assessment of rapid diagnostic tests for the detection of antibodies to hepatitis C virus, a tool for improving access to care. Clinical Microbiology and Infection. 2016;22(5):459. e1-. e6. doi: https://doi.org/10.1016/j.cmi.2016.01.009.
23. Khuroo MS, Khuroo NS, Khuroo MS. Diagnostic accuracy of point-of-care tests for hepatitis C virus infection: a systematic review and meta-analysis. PloS one. 2015;10(3):e0121450. doi: https://doi.org/10.1371/journal.pone.0121450.
24. Lee SR, Yearwood GD, Guillon GB, Kurtz LA, Fischl M, Friel T, et al. Evaluation of a rapid, point-of-care test device for the diagnosis of hepatitis C infection. J Clin Virol. 2010;48(1):15-7. Epub 20100401. doi: 10.1016/j.jcv.2010.02.018. PubMed PMID: 20362493.
25. World Health Organization. WHO Prequalification of In Vitro Diagnostics: Public Report. Product: OraQUick HCV Rapid Antibody Test Kit. WHO, 2017 WHO reference number: PQDx 0244-055-00.
26. Carvalho-Gomes ÂDc, Cubells ADc, Pallarés CDc, Hontangas VI, Conde IDc, Di Maira TI, et al. A population-based screening for hepatitis C antibodies and active infection using a point-of-care test in a low prevalence area. PLOS ONE [Internet]. 2020; 15(2).
27. Caballería L, Pera G, Bernad J, Canut S, Navarro E, Bruguera M. Strategies for the detection of hepatitis C viral infection in the general population. Revista clinica espanola. 2014;214(5):242-6. doi: 10.1016/j.rce.2014.01.024.
28. Niessen W, Benne R, van Zeijl J, van der Have J, Broer J. Is het schriftelijk oproepen van immigranten voor hepatitis B-onderzoek effectief? / IB 04-2013 2013. Jaargang 24, nummer 4:[Infectieziekten Bulletin]. Available from: https://www.rivm.nl/weblog/is-schriftelijk-oproepen-van-immigranten-voor-hepatitis-b-onderzoek-effectief-ib-04-2013.
29. Richter C, Ter Beest G, Gisolf E, Van Bentum P, Waegemaekers C, Swanink C, et al. Screening for chronic hepatitis B and C in migrants from Afghanistan, Iran, Iraq, the former Soviet Republics, and Vietnam in the Arnhem region, The Netherlands. Epidemiology & Infection. 2014;142(10):2140-6. doi: https://doi.org/10.1017/S0950268813003415.

30. Rossi C, Shrier I, Marshall L, Cnossen S, Schwartzman K, Klein MB, et al. Seroprevalence of chronic hepatitis B virus infection and prior immunity in immigrants and refugees: a systematic review and meta-analysis. 2012. doi: https://doi.org/10.1371/journal.pone.0044611.
31. Lee C, Emeto TI, Walsh N. Prevalence of hepatitis B virus amongst refugees, asylum seekers and internally displaced persons in low- and middle-income countries: A systematic review. Journal of viral hepatitis. 2023;30(1):4-18. doi: 10.1111/jvh.13770.
32. Alassad A, Al Rahwanji MJ, Yousfan A, Al Moualem S, Farhat A, Youssef LA. Seroprevalence and trends of hepatitis B virus, hepatitis C virus and human immunodeficiency virus in Syrian blood donors at Damascus University Blood Center between 2004 and 2021. Frontiers in Public Health. 2023;11. doi: 10.3389/fpubh.2023.1174638.
33. Freidl GS, Tostmann A, Curvers M, Ruijs WLM, Smits G, Schepp R, et al. Immunity against measles, mumps, rubella, varicella, diphtheria, tetanus, polio, hepatitis A and hepatitis B among adult asylum seekers in the Netherlands, 2016. Vaccine. 2018;36(12):1664-72. doi: 10.1016/j.vaccine.2018.01.079.
34. Duracinsky M, Thonon F, Bun S, Ben Nasr I, Dara AF, Lakhdari S, et al. Good acceptability of HIV, HBV, and HCV screening during immigration medical check-up amongst migrants in France in the STRADA study. PLoS One. 2020;15(6):e0235260. Epub 20200626. doi: 10.1371/journal.pone.0235260. PubMed PMID: 32589652; PubMed Central PMCID: PMCPMC7319329.
35. Lebano A, Hamed S, Bradby H, Gil-Salmerón A, Durá-Ferrandis E, Garcés-Ferrer J, et al. Migrants' and refugees' health status and healthcare in Europe: a scoping literature review. BMC Public Health. 2020;20(1):1039. doi: 10.1186/s12889-020-08749-8.
36. Bottero J, Boyd A, Gozlan J, Carrat F, Nau J, Pauti M-D, et al., editors. Simultaneous human immunodeficiency virus-hepatitis B-hepatitis C point-of-care tests improve outcomes in linkage-to-care: results of a randomized control trial in persons without healthcare coverage. Open forum infectious diseases; 2015: Oxford University Press.

List of abbreviations

HCC	Hepatocellular carcinoma
HBV	Hepatitis B virus
HCV	Hepatitis C virus
WHO	World Health Organization
DAA	Direct-acting antiviral agents
RIVM	The Dutch National Institute for Public Health and the Environment
PHS	Public Health Service
MEC-U	Medical Research Ethics Committees United
WMO	The Dutch Medical Research Involving Human Subjects Act
POCT	Point-of-care testing
GP	General practitioner

HBsAg	Hepatitis B surface antigen
Anti-HCV	Hepatitis C virus antibodies
FDA	Food and Drug Administration
CE	Conformité Européenne
MUMC+	Maastricht University Medical Centre
EDTA	Ethylenediaminetetraacetic acid
SPSS	The Statistical Package for the Social Sciences
HIV	Human immunodeficiency virus
