# Supplementary material for: Reaching Syrian migrants through Dutch municipal registries for hepatitis B and C point-of-care testing
Source: PLoS One. 2025 Jan 17;20(1):e0316726. doi: 10.1371/journal.pone.0316726 (PMC11741604; doi:10.1371/journal.pone.0316726)
Supplement: Attachment — Submitted filename: Comments_Moonen_etal_PONE-D-23-37923-febr-2024.doc [file pone.0316726.s001.doc]

February 20, 2024

Manuscript PONE-D-23-37923

Comments on manuscript:

‘Reaching Syrian migrants through Dutch municipal registries for hepatitis B and C point-of-care testing’

Moonen et al.

This well written manuscript describes a study among Syrian migrants in two Dutch cities. The aim was to develop an outreach strategy to screen them by measuring antibodies in the blood, against hepatitis B virus (HBV) and hepatitis C virus (HCV). The Syrian migrants were chosen because it was suspected that these infections were more highly prevalent than among other Dutch residents.

I have some minor comments to further improve the manuscript.

**Minor comments**

In the introduction two aims are stated (lines 74-75): 1. how to best reach the Syrian migrants in two Dutch municipalities and 2. To identify HBV and HCV infections in these migrants.

For the second aim I suggest to add everywhere in the manuscript that finger prick blood is used next to using a POCT since these two are not coupled and both are deviant from routine testing settings.

Abstract

Line 26: please add ‘(POCT)’ after ‘testing’, since this abbreviation is used later in line 32.

It is also good to add ‘using finger prick blood’ after ‘POCT’.

Multifaceted strategy

Line 127: since there are very many POCT on the market, I suggest to replace ‘the’ by ‘a’ before ‘point-of-care testing’.

Results

Legend of Fig 2. (line 220): I suggest to add ‘and test results’

Line 234: Please provide some more information on ‘short turnaround time’: what was the median time and range in minutes?

Discussion

Line 300: please adjust and do not start a new sentence. So ‘was made, for example….’.

Line 303: please adjust to: ‘non-participants’.

Line 326: Please replace ‘whom’ by ‘who’.

Line 332: Please adjust to ‘successful’.

Limitations

Although the DETERMINE	test for HBsAg and the OraQuick assay for HCV antibody detection are both stated to have a very high sensitivity, would you still consider using these in future settings on finger prick blood?

Also, I think it is a pity that no tests seem to have been performed concomittantly on regular EDTA blood samples (as was described to be performed in case confirmation was needed) and in the finger prick blood samples with the POCTs, to have an impression of the actual validity of the high sensitivity of these POCTs in this setting.
